# Infiltrating myeloid cell diversity determines oncological characteristics and clinical outcomes in breast cancer

**DOI:** 10.1186/s13058-023-01669-6

**Published:** 2023-06-07

**Authors:** Chenxuan Yang, Jiaxiang Liu, Shuangtao Zhao, Qingyao Shang, Fei Ren, Kexin Feng, Ruixuan Zhang, Xiyu Kang, Xin Wang, Xiang Wang

**Affiliations:** 1 Department of Breast Surgical Oncology, National Cancer Center/National Clinical Research Center for Cancer/Cancer Hospital, Chinese Academy of Medical Sciences and Peking Union Medical College, No.17 Panjiayuan Nanli, Chaoyang District, Beijing, 100021 China; 2 Department of Thoracic Surgery, Beijing Tuberculosis and Thoracic Tumor Research Institute/Beijing Chest Hospital, Capital Medical University, Beijing, China; 3 Peking Union Medical College, Chinese Academy of Medical Sciences and Peking Union Medical College, Beijing, China

**Keywords:** Breast cancer, Tumor microenvironment, Myeloid cells, Immunotherapy

## Abstract

**Background:**

Breast cancer presents as one of the top health threats to women around the world. Myeloid cells are the most abundant cells and the major immune coordinator in breast cancer tumor microenvironment (TME), target therapies that harness the anti-tumor potential of myeloid cells are currently being evaluated in clinical trials. However, the landscape and dynamic transition of myeloid cells in breast cancer TME are still largely unknown.

**Methods:**

Myeloid cells were characterized in the single-cell data and extracted with a deconvolution algorithm to be assessed in bulk-sequencing data. We used the Shannon index to describe the diversity of infiltrating myeloid cells. A 5-gene surrogate scoring system was then constructed and evaluated to infer the myeloid cell diversity in a clinically feasible manner.

**Results:**

We dissected the breast cancer infiltrating myeloid cells into 15 subgroups including macrophages, dendritic cells (DCs), and monocytes. Mac_CCL4 had the highest angiogenic activity, Mac_APOE and Mac_CXCL10 were highly active in cytokine secretion, and the DCs had upregulated antigen presentation pathways. The infiltrating myeloid diversity was calculated in the deconvoluted bulk-sequencing data, and we found that higher myeloid diversity was robustly associated with more favorable clinical outcomes, higher neoadjuvant therapy responses, and a higher rate of somatic mutations. We then used machine learning methods to perform feature selection and reduction, which generated a clinical-friendly scoring system consisting of 5 genes (C3, CD27, GFPT2, GMFG, and HLA-DPB1) that could be used to predict clinical outcomes in breast cancer patients.

**Conclusions:**

Our study explored the heterogeneity and plasticity of breast cancer infiltrating myeloid cells. By using a novel combination of bioinformatic approaches, we proposed the myeloid diversity index as a new prognostic metric and constructed a clinically practical scoring system to guide future patient evaluation and risk stratification.

**Graphical Abstract:**

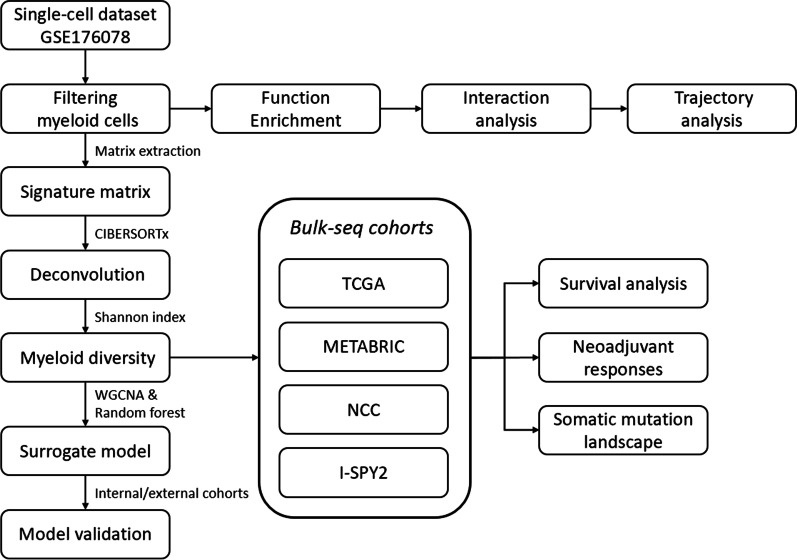

**Supplementary Information:**

The online version contains supplementary material available at 10.1186/s13058-023-01669-6.

## Introduction

Breast cancer (BRCA) is currently the most prevalent cancer and the leading cause of mortality or morbidity in women [1]. Traditional treatment modalities for BRCA include surgery, endocrinotherapy, chemotherapy, and radiotherapy, single or combination of these therapies are adopted according to the molecular subtype and stage of cancer [2]. In the past decade, T cell-based immunotherapies which either enhance endogenous T cell functions (immune checkpoint inhibitors (ICIs)) or infuse engineered T cells (CAR-T) have revolutionized treatment for several cancers [3]. However, the application of these T cell-based therapies in BRCA is still limited, partially due to the heterogeneity and a lack of T cell infiltration in certain subsets of patients [4]. The ineffectiveness of T cell-based immunotherapies in BRCA led investigators to attend to other immune components within the tumor microenvironment (TME) besides the cytotoxic cells. Of these components, myeloid cells are the most abundant, and various studies have underscored the importance of myeloid cells in modulating anti-tumor immunity, drug resistance, and metastasis [5, 6].

The myeloid lineage represents a broad group of cells each with distinct functions, major subgroups of myeloid cells include macrophages, dendritic cells (DCs), monocytes, and granulocytes. Most of the myeloid infiltrations in TME are derived from circulatory myeloid progenitors that originate from the bone marrow, while certain subsets of tissue-resident myeloid cells also contribute to the composition of the TME [7]. Different types of myeloid cells exert different influences on tumor progression: Macrophages are actively involved in antigen presentation, cell debris scavenging, cytokine secretion, and various other processes which can be either anti-tumor or pro-tumor, depending on the context. Previously dichotomized into either pro-inflammatory (M1) or anti-inflammatory (M2), it is now accepted that the complexity of macrophage subtypes is better characterized by an overlapping continuum of marker genes based on unbiased sequencing techniques instead of a binary categorization [8]. DCs are specialized antigen-presenting cells that play important roles in priming and activating anti-tumor T cell responses, they can be broadly grouped into conventional DCs (cDCs) and interferon-secreting plasmacytoid DCs (pDCs) [9]. Monocytes and granulocytes are usually less abundant, but they also have a profound impact on TME by initiating inflammatory responses and expressing inhibitory signals. Notably, certain subsets of immature monocytic and granulocytic cells can differentiate into myeloid-derived suppressor cells (MDSCs), which predominantly induce immunosuppression and promote tumor progression [10, 11].

Previous studies have implicated the importance of myeloid cells in the formation and shifting of the TME in different cancers, but these studies are often conducted with in vivo models, and the clinical characteristics of the diverse myeloid component in BRCA are still yet to be determined [12–14]. With the advancement of single-cell sequencing, it is now possible to depict the landscape of TME at a cellular level. Indeed, several single-cell studies have highlighted the indispensable role of myeloid cells in shaping the TME. However, most of single-cell-based studies focused on the phenotype and clinical significance of a certain subtype of myeloid cells, while the diverse yet interrelated network of different myeloid subtypes is rarely examined from a broader perspective [14, 15].

To fill this gap, the current study uses published and newly generated transcriptomic data to comprehensively dissect the infiltrating myeloid landscape in BRCA, characterize their phenotypic features, and elucidate their interactions. By using a combination of deconvolution algorithms and an ecological diversity index, we demonstrate the clinical implication of infiltrating myeloid diversity in the context of bulk-sequencing and propose a practical clinical index for evaluating myeloid infiltration in BRCA patients.

Our study provides novel insights to uncover the complexity of myeloid cells in BRCA and serves as a new resource for further development of myeloid-based therapies.

## Results

### Single-cell dissection of breast cancer infiltrating myeloid cells

**Fig. 1:**
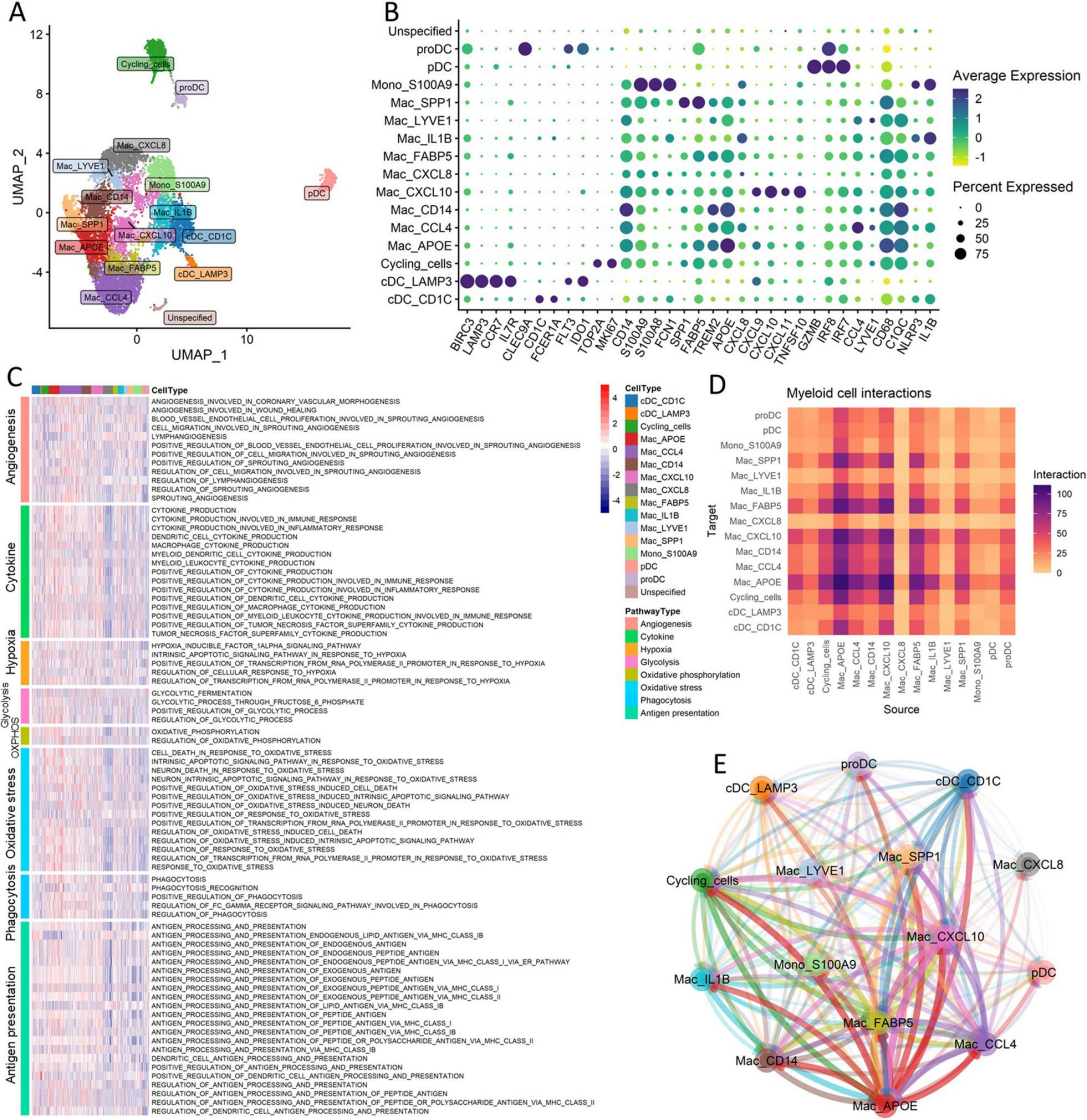
**A** Projection of different subgroups of breast cancer infiltrating myeloid cells in 2-dimensional UMAP space (axes represent dimensions, colors represent different cell subgroups). **B** Dot-plot of representative marker genes of each myeloid subgroup (*y* axis represents cell subgroups, *x* axis represents different genes, colors represent the average expression level of each gene, dot-size represents the expression percentage in each cell subtype). **C** Heatmap of single-sample gene set enrichment analysis (ssGSEA) score of major myeloid pathways in each subgroup (*y* axis represents the different pathways with breaks for major pathway types, *x* axis represents different cell subgroups, colors represent the enrichment scores (Red: high score; Blue: Low score)). **D** Interaction intensities between each myeloid subgroup as measured by interacting receptor pairs (*y* axis represents the receiver (target) of interactions, *x* axis represents the donor (source) of interactions). **E** Graph-based arrangement of different myeloid subgroups according to their interacting centricity (colors represent different cell subtypes, line weight represents the intensity of interactions)

Myeloid cells from single-cell RNA sequencing data were identified as clusters that highly express conventional myeloid markers, some of the markers used were CD68, C1QC, and FCN1. A total of 9170 myeloid cells were extracted from all cells, which were further clustered at a resolution of 1.0 into 15 subgroups (unspecified group not included) (Fig. 1A). We then used a curated list of marker genes to annotate the subgroups. Macrophages, characterized by CD68 and C1QC expression, formed the majority of myeloid cells, containing 9 subgroups. Monocytes were annotated according to elevated expression of S100A8 and S100A9, conventional dendritic cells (cDCs) were labeled by the characteristic expression of LAMP3, CD1C, and plasmacytoid dendritic cells (pDCs) were labeled by IRF7 and IRF8. Another group of cells expressing markers of both cDC and pDC were annotated as progenitor dendritic cells (proDC). Additionally, cells with increased cell-cycle-related gene expression such as MKI67 and TOP2A were deemed cycling cells (Fig. 1B).

Since myeloid cells are highly diversified in biological functions, we performed functional enrichment analysis to reveal their characteristics. Major myeloid functions were broken down into 8 categories covering angiogenesis, cytokine production, phagocytosis, antigen presentation, glucose metabolism, oxidative stress, and hypoxia responses. Functional activity of each category was assessed through single-sample gene set enrichment analysis (ssGSEA) (Fig. 1C). We found that Mac_CCL4 was highly active for angiogenesis, while DCs showed an overall downregulation of angiogenic pathways. Mac_APOE and Mac_CXCL10 showed elevated cytokine production activity, yet Mac_CXCL8 was repressed for cytokine-related pathways. Interestingly, we found that Mac_CXCL8 showed decreased activity in most functional categories except for angiogenesis, suggesting that this group of cells might be in a state of terminal differentiation or dormancy. Conversely, Mac_APOE and Mac_CXCL10 showed increased glucose metabolism, oxidative stress responses, and phagocytotic activity, indicating that these two types of cells might be highly active and present as the central coordinators of innate immune responses. Monocytes, Mac_SPP1, and Mac_LYVE1 were deactivated in antigen presentation pathways, while the DCs, as expected, were the main population for antigen presentation.

To further elucidate the interactions between different myeloid components, we interrogated the cell–cell communication through the expression of various ligand-receptor pairs. Similar to the functional enrichment analysis, we found that Mac_APOE and Mac_CXCL10 were the most active communicators in the myeloid cells, but Mac_CXCL8 had a rather dormant phenotype (Fig. 1D). Mac_FABP5 and Mac_SPP1 were also actively involved in myeloid communications, both as target cell and source cell. Compared with the cross-interacting macrophages, the DCs had lesser communications with other myeloid cells, possibly due to their functions as antigen-presenting cells outside the scope of myeloid cells. By arranging the cells depending on their connectivity in a graph-based algorithm, we found that the aforementioned Mac_APOE, Mac_CXCL10, Mac_FABP5, and Mac_CCL4 were located in close adjacency, while other macrophage subtypes and monocytes were distributed in a less interrelated manner. The DCs and Mac_CXCL8 were located at a further location, exhibiting a somewhat secluded behavior (Fig. 1E).

To understand the transition of infiltrating myeloid cells on a single-cell level, we used trajectory analysis to investigate the shifting of myeloid subtypes (Additional file 1: Fig. S1A and B). We showed that cells with high activity in angiogenesis were located at a later pseudotime stage, while cells with higher cytokine secretion activity appeared at an earlier stage, consistent with the previous paradigm that tumor-associated macrophages shift from inflammation-mediating to an angiogenesis-based wound healing phenotype. Supportive of these findings, we observed elevated expressions of angiogenic genes such as VEGFB, TGFB1, and IGF1 as pseudotime increased (Additional file 1: Fig. S1C, upper panel). On the contrary, Inflammatory mediators such as NLRP3 and IL1B decreased over pseudotime, whereas the anti-inflammatory cytokine IL10 showed an opposite trend (Additional file 1: Fig. S1C, mid panel). Furthermore, we observed increased expression of several myeloid-based immunotherapy targets over the pseudotime trajectory, including CSF1R, CD40, and SIRPA (Additional file 1: Fig. S1C, bottom panel). Transcription factor and protein kinase analysis identified key regulatory pathways driving the phenotypic transition of myeloid cells over pseudotime (Additional file 1: Fig. S1D and E).

### Myeloid infiltration diversity robustly determines clinical outcomes

**Fig. 2:**
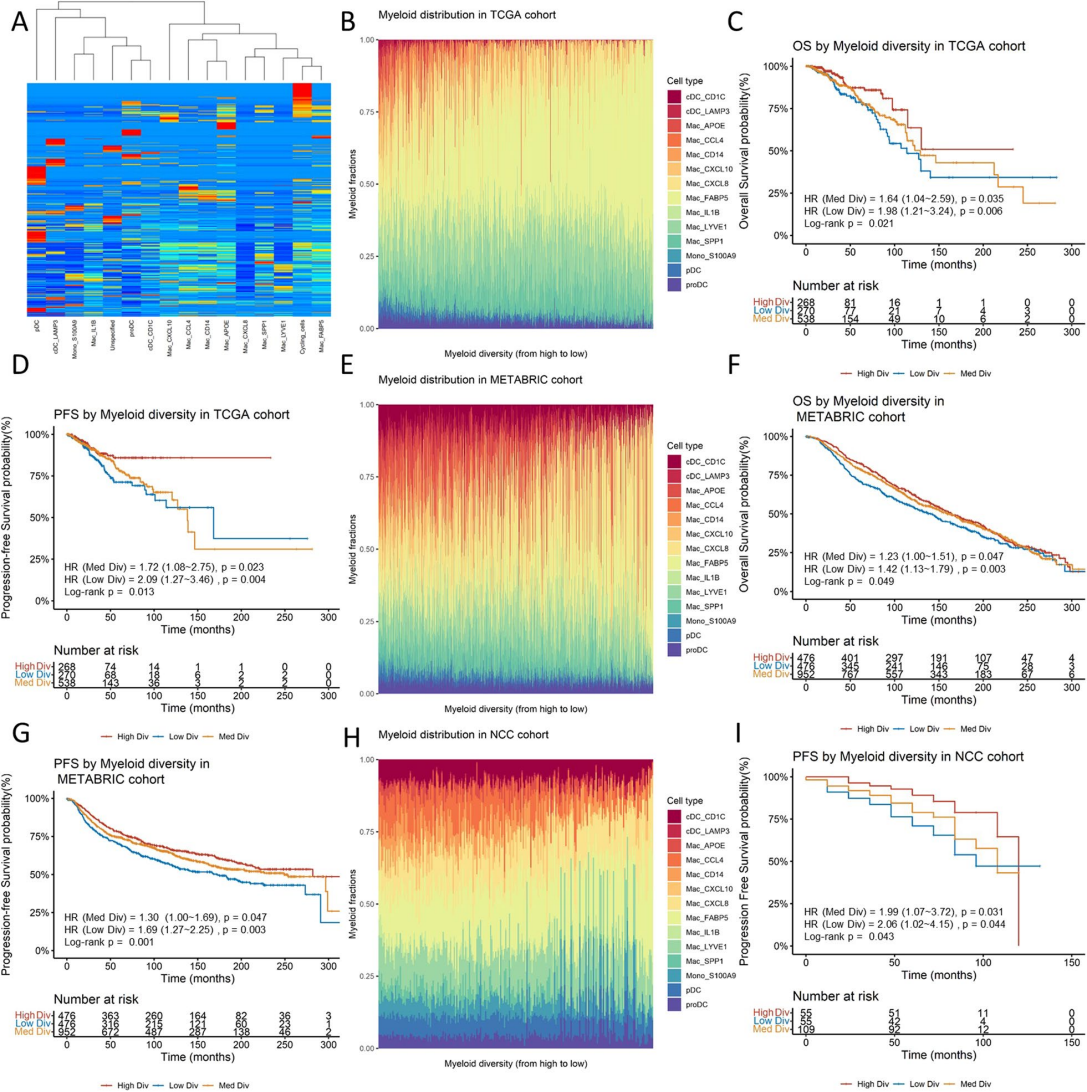
**A** Clustered signature matrix of breast cancer infiltrating myeloid cells derived from single-cell analysis (*y* axis represents different genes, *x* axis represents cell subgroups, color represents gene expression levels (red: high expression; blue: low expression)). **B** Infiltrating myeloid cell distribution in the TCGA cohort (*y* axis represents cell percentage in different colors, *x* axis represents myeloid diversity arranged in a decreasing manner). **C**, **D** Kaplan–Meier plot of survival analysis for patients with different levels of myeloid diversity in the TCGA cohort (OS: overall survival, PFS: progression-free survival, colors represent different groups). **E** Infiltrating myeloid cell distribution in the METABRIC cohort. **F**, **G** Kaplan–Meier plot of survival analysis for patients with different levels of myeloid diversity in the METABRIC cohort. **H** Infiltrating myeloid cell distribution in the NCC cohort. **I** Kaplan–Meier plot of survival analysis for patients with different levels of myeloid diversity in the NCC cohort

Myeloid infiltration in breast cancer is highly dynamic, studies focusing on a single subtype might not reflect the full picture of myeloid biology in the clinical setting. Therefore, we aimed to use the diversity indices as quantifiable metrics to investigate the diversity of different types of myeloid infiltration. Three frequently used diversity indices were evaluated in our analysis, namely the Shannon index, Gini-Simpson index and the Pielou index[16]. Our analysis showed that the three indices were highly correlated with each other and generated comparable results in subsequent analyses (Additional file 1: Fig. S2 and Table S1). We therefore chose to use Shannon index to represent the myeloid diversity since it assigns greater weight to rare species which could exert a significant impact in immunological settings. The signature matrix of the 15 myeloid subgroups were extracted and deconvoluted using CIBERSORTx to infer the infiltration abundance in bulk-seq data (Fig. 2A). In the TCGA cohort, the calculated myeloid diversity index appropriately reflected the heterogeneity of myeloid cell infiltration, the DC and monocyte infiltration reduced to a marginal amount as the diversity index increased, while macrophages became the dominant infiltrating component (Fig. 2B). We found that the Luminal-B subtype had significantly lower diversity than Luminal-A and HER2-enriched subtypes. The basal-like subtype, being the most heterogeneous subtype, showed high variation in the distribution of the diversity index (Additional file 1: Fig. S3A). Since it is generally considered that tumor microenvironments with highly diversified immune infiltration could be beneficial to patients, we tested whether the myeloid diversity index could determine clinical outcomes. We found that higher myeloid diversity was significantly associated with better overall survival (OS) rate (HR = 0.1619) and progression-free survival (PFS) rate (HR = 0.2369) (Table 1). Multivariate Cox regression revealed that the prognostic power of myeloid diversity was independent of demographic status and the breast cancer subtypes (Additional file 1: Table S2 and S3). Comparable results were obtained by stratifying the patients using upper and lower quantiles and comparing with the log-rank test (Fig. 2C and D). To test the robustness of the diversity index in predicting prognosis, we validated these analyses using the METABRIC cohort and a cohort from our facility (NCC cohort). The distributions of myeloid diversity index were compared between cohorts (Additional file 1: Fig. S3B). We found that the changes in myeloid infiltration along the diversity index showed similarity in all three cohorts, as exemplified by the increasing dominance of certain cell types, such as Mac_FABP5 (Fig. 2E and H). The survival analysis showed that patients with higher myeloid diversity had more favorable OS (HR = 0.6025) and PFS (HR = 0.4897) in the METABRIC cohort as well as in the NCC cohort (HR for PFS = 0.2031) (Fig. 2F, G and I, Table 1). Multivariate Cox regression revealed that myeloid diversity was prognostic independent of demographic status and the breast cancer subtypes in the two cohorts (Additional file 1: Table S4–7). To compare the predictive ability of the myeloid diversity index with individual myeloid cell types, we then conducted univariate Cox regression analyses. Although certain myeloid cell types also showed prognostic significance in specific cohorts, the myeloid diversity index demonstrated better overall robustness across all cohorts (Additional file 1: Table 8). In our next step, we sought to externally validate the prognostic significance of the myeloid diversity index. To this end, we conducted the same analytic pipelines for myeloid diversity in prostate cancer, another solid tumor driven by sex hormone. Specifically, we extracted the signature matrix of infiltrating myeloid cells in prostate cancer using single-cell transcriptomic data from Wouter & Matan et al.'s work [17], and subsequently subjected the signature matrix to deconvolution and Shannon index calculation. The prognostic value of the myeloid diversity index was then assessed in the TCGA-PRAD cohort. The results showed that the myeloid diversity index also holds prognostic value in prostate cancer (Additional file 1: Table S9 and Fig. S4).

**Table 1:** Univariate Cox regression results of the myeloid diversity index and surrogate index in three cohorts

Term	Hazard ratio (HR)	Lower 95% CI	Upper 95% CI	*p*-value	Survival data	Cohort
Diversity index	0.1619	0.0528	0.4961	0.0014	OS	TCGA
Diversity index	0.2369	0.0763	0.7356	0.0127	PFS	TCGA
Diversity index	0.6025	0.4215	0.8612	0.0054	OS	METABRIC
Diversity index	0.4897	0.3181	0.7539	0.0012	PFS	METABRIC
Diversity index	0.2031	0.0547	0.7543	0.0173	PFS	NCC
Surrogate index	0.0054	0.0001	0.5133	0.0246	PFS	TCGA
Surrogate index	0.0249	0.0012	0.5246	0.0176	PFS	NCC
Surrogate index	0.2899	0.1104	0.7616	0.0120	PFS	METABRIC

**Fig. 3:**
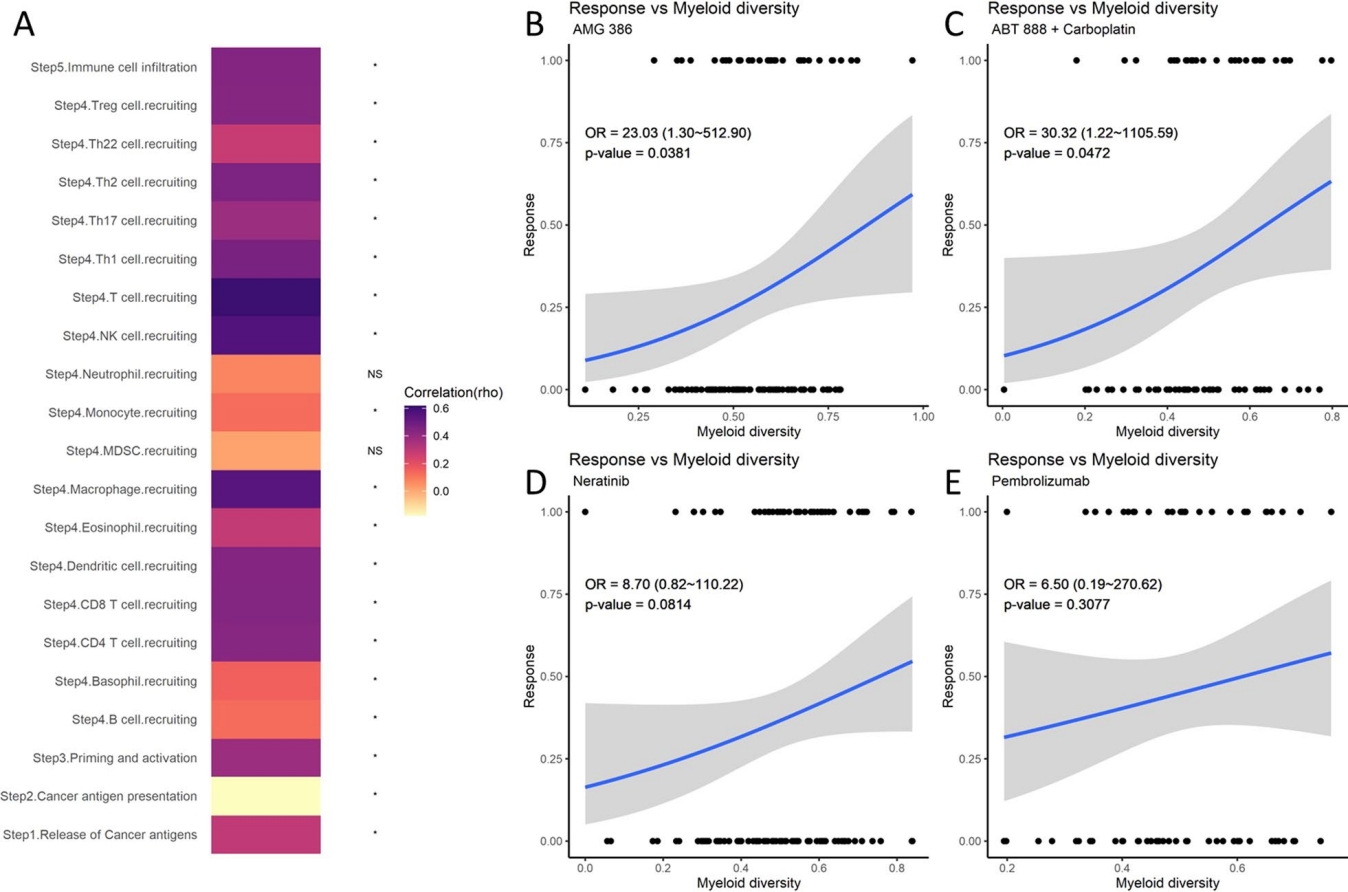
**A** Heatmap of correlation between myeloid diversity and the activities of critical steps in anti-tumor immunity (colors represent the correlation coefficient, asterisks: *p* < 0.05, NS: non-significant). **B–E** Logistic regression of myeloid diversity to response rates of neoadjuvant therapies (AMG 386, ABT888/Carboplatin, Neratinib, and Pembrolizumab) from the I-SPY2 study (*y* axis represents the neoadjuvant responses of patients in the trials, *x* axis represents the normalized myeloid diversity index, OR: odds ratio calculated from the logistic regressions)

Apart from survival analysis, we also aimed to investigate the relationship of myeloid diversity with other potential clinical features. We first studied whether the diversity index is associated with anti-tumor immunity and found that the diversity index was positively correlated to most of the anti-tumor immunity steps, especially in macrophage recruiting and T cell recruiting (Fig. 3A). Next, we performed logistic regression to analyze the association of myeloid diversity with neoadjuvant therapy responses using the RNA-seq data from I-SPY2 trials. From the 10 arms from the trials, we found the responses of AMG-386 (trebananib) and ABT-888 (veliparib)/carboplatin showed a marginal yet significant fit with the diversity index in the regression model (*p* = 0.038 and 0.047, respectively) (Fig. 3B and C), while the fit for neratinib response was non-significant (*p* = 0.081) (Fig. 3D). Interestingly, we found that responses to anti-PD1 therapy (pembrolizumab) were not significantly associated with myeloid diversity (Fig. 3E), suggesting potential independent mechanisms of T cell-based and myeloid-based therapies.

### Myeloid diversity was associated with different somatic mutation landscapes.

**Fig. 4:**
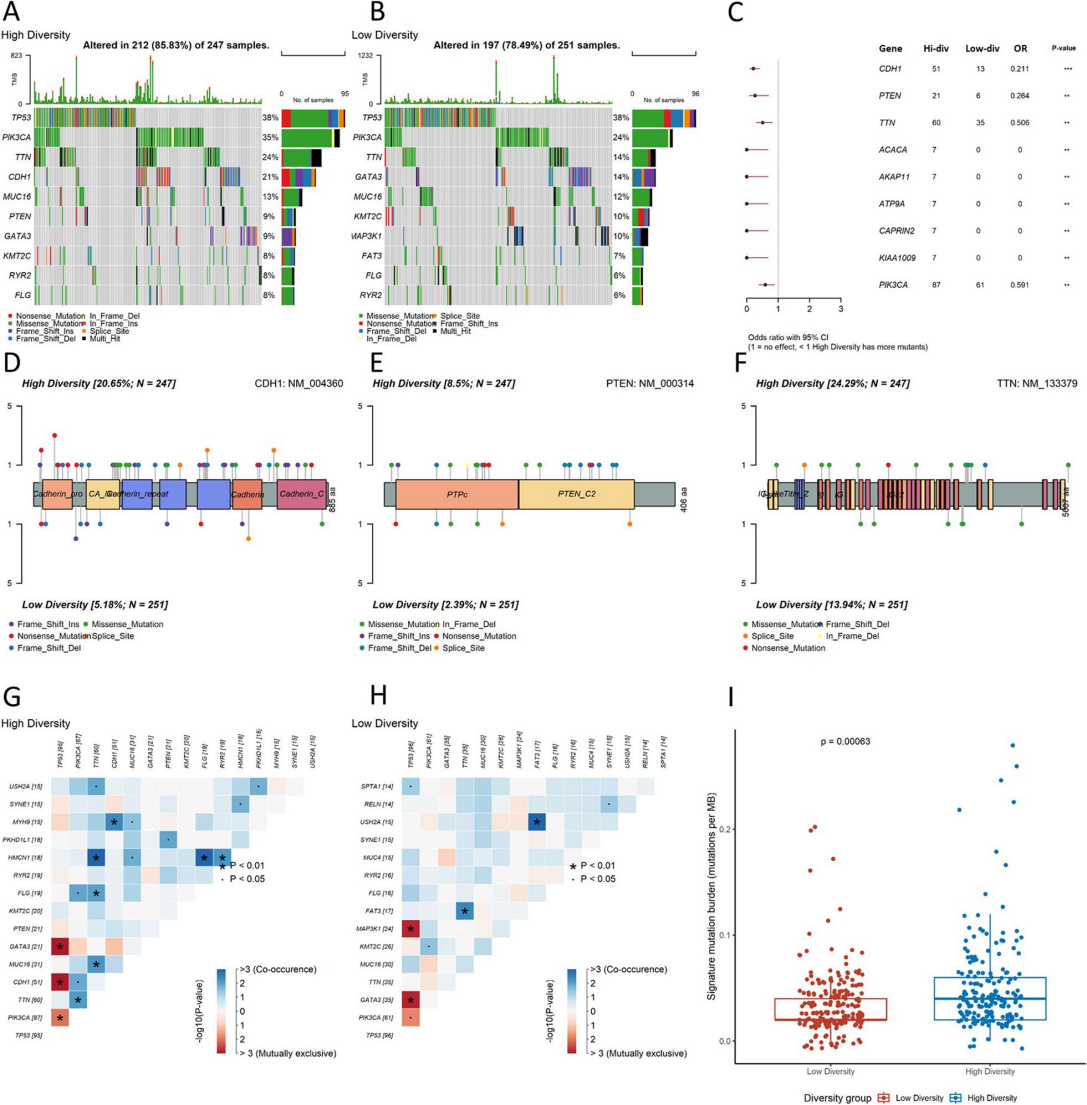
**A**, **B** Heatmap of top 10 most frequent somatic mutations in high and low myeloid diversity groups of patients from the TCGA cohort (*y* axis represents the top 10 highly mutated genes, *x* axis represents different patients, colors represent different types of somatic mutations, tumor mutation burdens were presented in a bar graph above the heatmap). **C** Forest plots of somatic mutations that were differentially distributed in high and low myeloid diversity groups, *p*-values were calculated from chi-square tests (*: *p* < 0.05, **: *p* < 0.001, ***: *p* < 0.0001). **D–F** Schematic plot showing highly mutated domains in the protein structures of CDH1, PTEN, and TTN (colors of the lollipops represent different types of somatic mutations). **G**, **H** Correlation heatmap of co-occurrence and mutual-exclusive mutations in high and low myeloid diversity groups (axes represent different genes, colors represent the correlations (blue: co-occurring; red: mutual-exclusive)). **I** Boxplot of the signature tumor mutation burden between high and low diversity groups (colors represent different groups (red: low diversity group; blue: high diversity group), the Wilcoxon test was used to compare groups)

Previously it has been proposed that somatic mutations could generate neoantigens for antigen presenting cells such as myeloid cells. We therefore asked whether the myeloid diversity index is associated with different somatic mutation landscapes. By performing somatic mutation analysis, we found that TP53, PIK3CA, and TTN were the most prevalent in both high and low diversity groups (Fig. 4A and B), although the occurrence of PIK3CA (OR = 0.591) and TTN (OR = 0.506) mutations were significantly higher in high diversity group (Fig. 4C). From the less prevalent mutated genes, we found that the occurrences of CDH1 (OR = 0.211), PTEN (OR = 0.264), ACACA, AKAP11, ATP9A, CAPRIN2, KIAA1009 were significantly higher in high diversity group. To further understand the mutational patterns of these genes, we conducted mutational site analysis to depict the protein structures along with mutation sites for CDH1, PTEN, and TTN, and PIK3CA. CDH1 had a fourfold increase in mutation rate in the high diversity group compared with the low diversity group, the mutation sites were evenly distributed across the protein structure (Fig. 4D). PTEN, however, had mutation sites more frequently located at the C2 domain, most of which were frame-shift deletions (Fig. 4E). TTN and PIK3CA showed a high rate of missense mutation at various sites in the high diversity group, while other types of mutations were less common in these two genes (Fig. 4F and Additional file 1: Fig. 5A). Next, we analyzed the co-occurrence of mutations in the high and low diversity groups. The most prevalent co-mutated genes in the high diversity group included HMCN1-TTN, HMCN1-FLG, and MYH9-CDH1. We also found that mutations of TTN, CDH1 and PIK3CA had a rather high probability of co-occurrence, which is largely in line with previous analyses (Fig. 4G). Moreover, we observed a significantly high rate of mutual exclusions of TP53 mutations to GATA3, CDH1, and PIK3CA mutations. In the low diversity group, we found that FAT3-TTN and FAT3-USH2A showed a high rate of co-occurrence (Fig. 4H). Similar to that in the high diversity group, TP53 mutations showed a high rate of mutual-exclusion to GATA3 and PIK3CA mutations, yet we also found a TP53-MAP3K1 mutual-exclusion which was not observed in the high diversity group. Tumor mutation burden (TMB) has recently been recognized as an important marker for the neoantigen production. We therefore performed TMB analysis by defining TMB as the number of somatic mutations per mega-base of genomic sequence. The overall TMB (mutations of all genes included) was similar between high and low diversity groups while slightly varied across subtypes (Additional file 1 Fig. 5B and C). However, we found that a signature TMB, which included the prevalent mutations from both diversity groups along with the significant interacting mutations, were significantly higher in the high-diversity group and showed no prevalence among subtypes (Fig. 4I, Additional file 1: Fig. 5D and E).

### Construction of a surrogate myeloid diversity index for clinical application

**Fig. 5:**
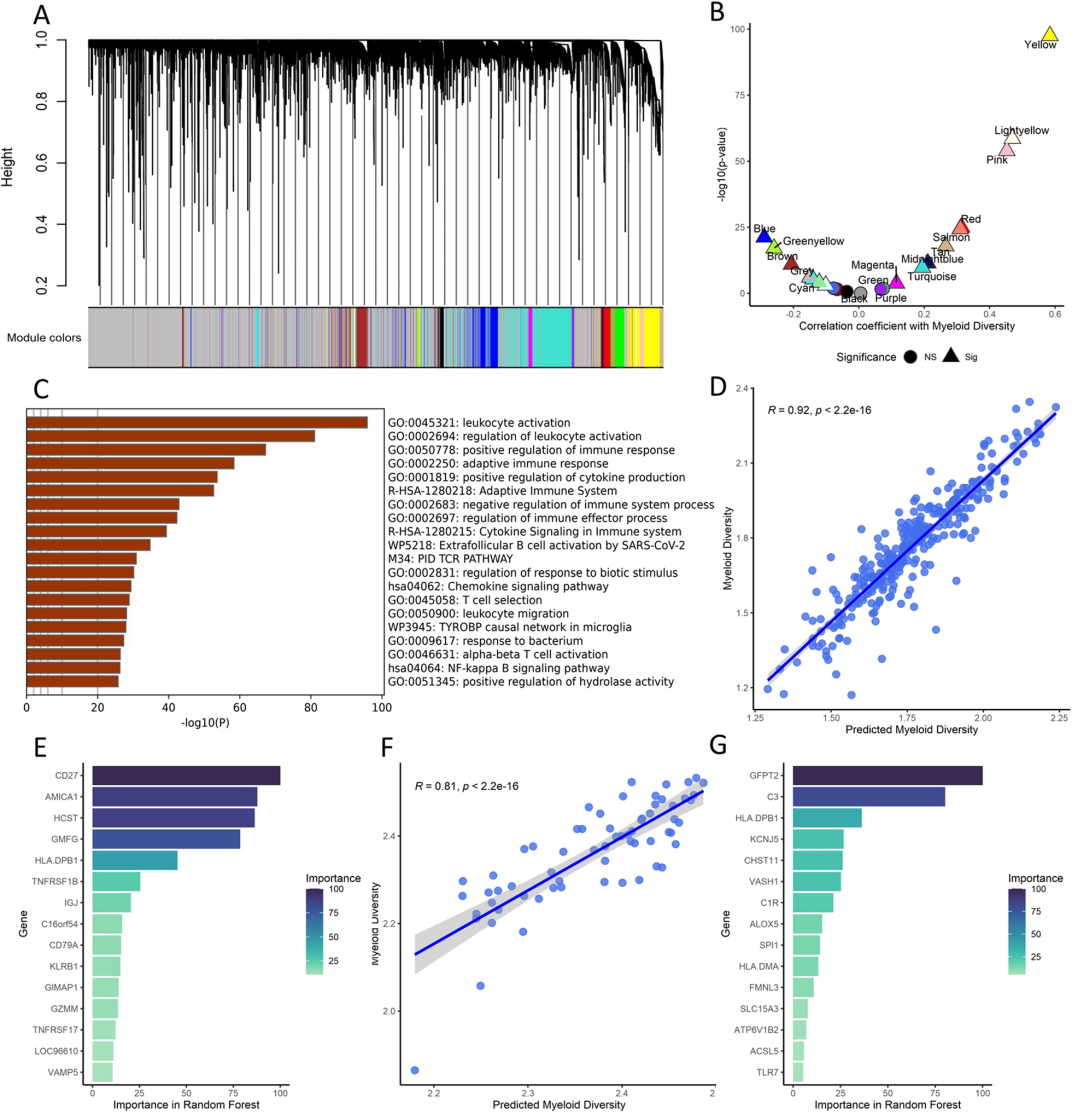
**A** Dendrogram of gene modules identified from weighted gene co-expression network analysis (WGCNA) (*x* axis represents different genes, colors represent different gene modules). **B** Correlation between the eigenvalue of each WGCNA module and the myeloid diversity (*y* axis represents log-transformed *p* values, *x* axis represents correlation coefficients, colors represent different gene modules, shapes represent correlation significance (triangle: significant; round: non-significant)). **C** Functional gene set enrichment of yellow module genes (*y* axis represents different pathways, *x* axis represents log-transformed *p* values). **D** Correlation between the random forest-predicted myeloid diversity and the original myeloid diversity in the TCGA cohort. **E** Top 15 contributing genes in the random forest model from the TCGA cohort, arranged by their relative importance (*y* axis represents different genes, *x* axis represents the importance in the random forest model). **F** Correlation between the random forest-predicted myeloid diversity and the original myeloid diversity in the NCC cohort. **E** Top 15 contributing genes in the random forest model from the NCC cohort, arranged by their relative importance

Although the myeloid diversity index calculated above showed decent predictive power and robustness for clinical outcomes of breast cancer, it was difficult to be applied to clinical practice. Therefore, we aimed to develop a surrogate myeloid diversity index using feature reduction methods. We first employed weighted gene co-expression network analysis (WGCNA) to generate 22 different gene modules from the TCGA cohort (Fig. 5A). The eigenvalue of each group was then extracted and compared with the myeloid diversity index for correlation. We found that the yellow module showed the highest correlation with myeloid diversity (*p* < 0.001) (Fig. 5B). The yellow module genes were then used to perform enrichment analysis, which showed that they were indeed highly related to leukocyte activity, immune responses, and cytokine secretion (Fig. 5C).

To further filter for an optimal number of genes for the surrogate model to reflect the myeloid diversity index, we used machine learning-based methods to perform a second feature reduction process. A random forest model was trained in the training dataset from the TCGA cohort with the yellow module genes. The model showed good fitting power as the predicted diversity index highly correlated with the actual diversity index in the testing dataset (Fig. 5D). We then calculated the importance of each gene to compare their contribution to the random forest model and found that CD27, AMICA1, HCST, GMFG, HLA-DPB1 were among the top contributing genes in the model (Fig. 5E). Similar model training and testing were conducted in the NCC cohort (Fig. 5F), and we found that HLA-DPB1, GFPT2 and C3 showed high contribution to the model (Fig. 5G).

**Fig. 6:**
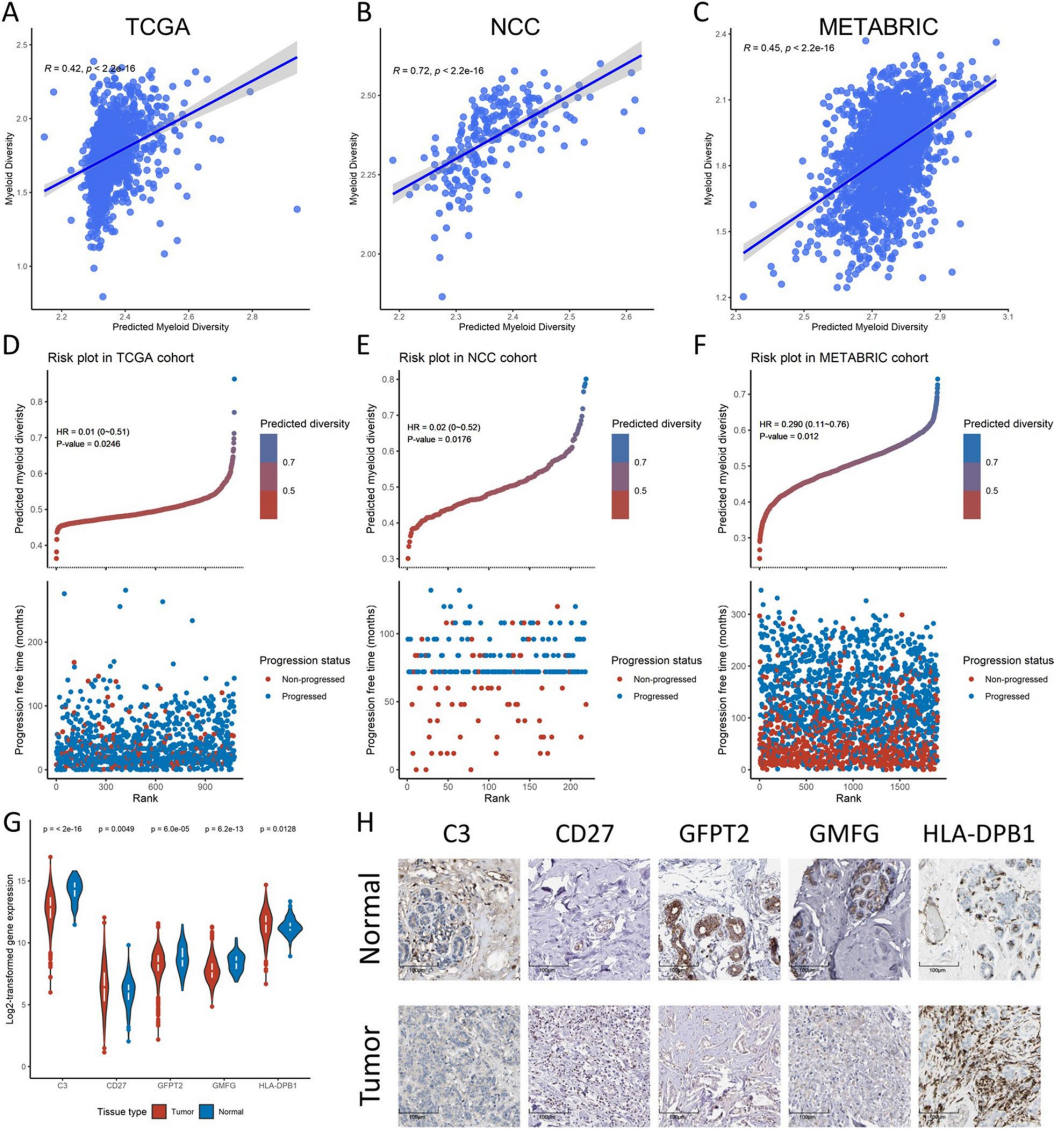
**A–C** correlation between the surrogate 5-gene model-predicted myeloid diversity and the original myeloid diversity in the TCGA, NCC, and METABRIC cohort. **D–F** Risk plots of surrogate myeloid diversity and their corresponding univariate cox regression results in the TCGA, NCC, and METABRIC cohort (*y* axis of top panel represents the predicted myeloid diversity, *y* axis of bottom panel represents the follow-up time, *x* axis represents different patients ranked by the predicted myeloid diversity, colors of top panel represent values of predicted myeloid diversity, colors of bottom panel represent progression status of patients). **G** Expression of the surrogate model genes in tumor and normal tissues, compared using transcriptomic data (*y* axis represents log-transformed expression of each gene, *x* axis represents different genes, colors represent tissue types). **H** Expression of the surrogate model genes in tumor and normal tissues, compared using immunohistochemistry (scale: 100 μm, target proteins were stained in brown)

The top contributing genes from both TCGA and NCC cohorts were then used to construct the surrogate model (Additional file 1: Table 10). Multi-collinearity was optimized by filtering out genes that were highly linear-correlated, and the final surrogate model was constructed with C3, CD27, GFPT2, GMFG, and HLA-DPB1. We tested the correlation of the predicted myeloid diversity from the surrogate model with the actual myeloid diversity index from the deconvolution algorithm and found that these two metrics were not only correlated in the TCGA and NCC cohorts from which they were derived (Fig. 6A and B), but also moderately correlated in the METABRIC cohort as an external validation (Fig. 6C). The Cox regression was performed, and we found that higher surrogate myeloid diversity index was significantly associated with better PFS rates in all three cohorts (Table 1, Fig. 6D-F), multivariate regression showed that the surrogate indices were prognostic independent of other demographic status (Additional file 1: Table S11–13). Finally, we analyzed whether the 5 genes in the model were differentially regulated in tumors compared with normal tissues. The mRNA expression showed that all five genes were differentially expressed in breast cancer: C3, GFPT, and GMFG were significantly down-regulated, while CD27 and HLA-DPB1 were significantly up-regulated (Fig. 6G). The immunohistochemistry staining from the THPA library also confirmed these findings, showing dysregulation of the 5 genes in breast cancer slides (Fig. 6H).

## Discussion

Myeloid cells are major components of the tumor microenvironment (TME) and play important roles in regulating both anti- and pro-tumor bioprocesses. We investigated the infiltrating myeloid cells from breast cancer (BRCA) patients using single-cell sequencing, defined 15 distinct subtypes of myeloid cells, and delineated their phenotypic characteristics. We employed a deconvolution algorithm and used the Shannon index to reflect the diversity of infiltrating myeloid cells. The index was assessed in determining clinical outcomes and a surrogate 5-gene model was developed to aid clinical practices.

Our analysis revealed several macrophage subtypes which highly resembled those identified in previous single-cell studies were also detected in our study: Mac_SPP1 had an elevated angiogenic signature and an overall pro-tumor activity, as reported by studies in several other cancers [18, 19]; Mac_APOE and Mac_FABP5 showed increased expression of marker genes including APOE, FABP5, and TREM2, which were indicated in lipid-associated macrophages. These macrophages are presumably important in managing lipid metabolism and closely interact with adipocytes which abound in the breast tissue [20]; Mac_LYVE1 showed a decreased capability of cytokine production and an anti-inflammatory phenotype. This coincides with a previous study that identified LYVE1hi macrophages as specific tissue-resident macrophages and showed that depletion of these macrophages resulted in an exacerbated inflammatory response [21]. Apart from these previously identified macrophages, we also annotated several macrophage subtypes using an established spectrum of macrophage-specific genes [8]. The Mac_IL1B and Mac_CXCL10 were active in inflammatory cytokine secretion and phagocytosis, resembling the so-called ‘M1’ macrophages, while the Mac_CCL4 were more active in regulating angiogenesis and showed highly activated oxidative stress pathways. The Mac_CXCL8 subgroup showed a somewhat suppressed phenotype in most macrophage-related functions except for angiogenesis, this group was also the least active in cell–cell interactions, suggesting a dormant and potentially pro-tumor role in the TME. Interestingly, we also identified a group of macrophages with high CD14 expression, which was deemed a maker of myeloid-derived suppressor cells (MDSCs) [11]. However, this group of cells also express APOE, C1QC, and TREM2, which were considered macrophage markers. We believe that these cells represent a functional state somewhere between MDSCs and mature macrophages which agrees with the idea that myeloid cells are in a continuous spectrum of differentiation instead of discreet states. Another major type of myeloid cell was the dendritic cells (DCs). Consistent with previous understanding of DCs, we found that all DC populations showed elevated antigen-presenting activities. The plasmacytoid DCs (pDCs) were especially suppressed in angiogenic pathways, suggesting a potentially anti-tumor role that could be harnessed for therapeutic development.

Apart from dissecting the infiltrating myeloid landscapes in BRCA, we also found that the diversity of infiltrating myeloid cells had unignorable implications in clinical and biological settings. The Shannon index was originally an ecological term to describe the diversity of species. We, for the first time, used the Shannon index to reflect infiltrating myeloid diversity in the TME and found that as diversity decreased, the infiltrating DCs gradually diminished while certain macrophages (mainly Mac_FABP5 and Mac_CXCL8) became the dominant myeloid cell. We speculate that this gradual loss of antigen-presenting cells and inflammatory cells represents the immunoediting process where the TME was tamed by the tumor and the anti-tumor immunity was overcome. Indeed, by using myeloid diversity as the main variable in survival analysis, we found that higher myeloid diversity was associated with better clinical outcomes and higher neoadjuvant response rates in 4 different cohorts. Somatic mutations were considered an important source of neoantigens, we asked whether the myeloid diversity was also associated with differences in somatic mutations. By interrogating the mutational profiles in the TCGA cohort, we found 9 genes that showed higher mutation rates in the high diversity group compared with the low diversity group. These results indicate that somatic mutation rate can be used to select patients who are potentially benefited from myeloid-based therapies and that mutations of these 9 genes might generate certain neoantigens to facilitate myeloid-mediated anti-tumor immunity more effectively than other genes.

Although the myeloid diversity index calculated from single-cell data and deconvolution algorithms were robust indicators of clinical outcomes, the massive data acquiring and calculating process made it unattainable in actual clinical settings. Therefore, we used stepwise feature selection methods to construct a practical model which maximally retained the information from the original myeloid diversity while minimizing the number of genes required. A 5-gene surrogate model was constructed which included C3, CD27, GFPT2, GMFG, and HLA-DPB1, all of which were important immune-regulating genes. The complement C3 is a crucial activator of phagocytosis, it was reported to regulate cancer metastasis [22]. CD27 belongs to the tumor necrosis factor (TNF) receptor superfamily which contributes to multiple downstream effects of TNF activation. The relationship between GFPT2 and breast cancer is largely unknown until a recent study reported that GFPT2 regulates macrophage mitochondrial fission to facilitate the phagocytosis of cancer cells [23]. GMFG was reported to modulate iron metabolism and TLR4 responses in macrophages which potentially benefit tumor clearance [24, 25]. Finally, HLA-DPB1 is part of the major histocompatibility complex II (MHCII), which plays key role in the antigen presentation of macrophages and DCs. As introduced above, the 5 genes largely covered the main functionalities of myeloid cells which makes them reliable candidates in the surrogate model to fit the original myeloid diversity. Indeed, the 5-gene surrogate model was predictive of clinical outcomes in 3 different cohorts, including an external validating cohort. However, it is important to note that while the surrogate model exhibited reasonable robustness across the three cohorts, its overall performance was not enough to meet the requirements for widespread implementation in clinical settings. We attribute this observation to the fact that the TCGA and NCC cohorts consisted of RNA sequencing data from different racial backgrounds, whereas the METABRIC cohort utilized microarray data, potentially introducing batch effects across platforms, and limiting the model's generalizability. Additionally, the performance of the model in the development cohorts was intentionally constrained to prevent overfitting and enhance its performance in external cohorts. Moving forward, we are committed to further enhancing the model in future research by exploring more advanced feature selection methods and regression algorithms to refine its predictive capabilities. We also believe further mechanistic studies focusing on these genes could yield valuable information and enhance our understanding of the model as well as the biology of tumor-infiltrating myeloid cells.

Our study was so far the first to propose a reliable and robust index to infer infiltrating myeloid diversity in BRCA. However, the study had certain limitations: (1) the single-cell sequencing dataset from which we extracted the myeloid signature matrix had a rather small sample number and limited genetical backgrounds. The model derived in this study, although showing decent robustness, could be outperformed by models derived from cohorts covering a more diverse cohort. (2) Although we analyzed the transition of myeloid cells on a pseudotime basis, it is still far from clear what the driving mechanism was that facilitated the differentiation of myeloid cells toward shaping a tumor-favoring environment, and whether this process can be reverted or diverted with therapeutical interventions. (3) The NCC cohort had a rather short follow-up time, and the overall survival status was not readily available for the current study. The survival analyses were therefore performed on different endpoints during model validation. (4) Our study provided HR values calculated from the diversity index in the Cox regression model, however, it should be noted that the interpretation of HR values across cohorts could be influenced by multiple factors, including the scaling of the index, the units of RNA expression, and the sequencing platforms used in each cohort. It would be important to take these factors into account when applying the surrogate model in future studies. (5) The current study was conducted mainly with bioinformatical methods, yet the biological significance of the myeloid diversity index and the 5 genes need to be tested via in vivo and in vitro experiments to elucidate their molecular mechanisms and aid the development of specified treatments.

In summary, our study used a novel combination of single-cell and bulk sequencing to establish a novel pipeline for analyzing tumor-infiltrating myeloid cells. We pictured the heterogeneity and plasticity of different myeloid cells in breast cancer, proposed the myeloid diversity index as a new prognostic metric, and developed a clinically practical model to guide future patient evaluation and stratification.

## Methods

### Single-cell sequencing data acquisition

The single-cell transcriptomic data was downloaded from the Gene Expression Omnibus under accession number GSE176078 [26]. The tissue preparation and sequencing process were as described in the original publication. Briefly, samples were surgically resected and dissociated with Human Tumor Dissociation Kit, and viability was assessed with Annexin V staining. The sequencing was performed using Chromium Single-Cell v2 3′ and 5′ Chemistry Library, Gel Bead, Multiplex, and Chip Kits. The raw data was then processed with Cell Ranger software from 10 × Genomics. Patient ethics were approved by the relevant committee, all downloaded data were de-identified.

### Single-cell data analysis

The counts, barcodes, and features matrix were loaded and analyzed with Seurat v4.0.5 in R v4.1.0 using the standard pipeline. Briefly, all the data were normalized before selecting highly variable genes for principal component analysis (PCA). Then, a shared-nearest-neighbors (SNN) graph was created based on the PCA results from these highly variable genes, after which the cells were clustered with a modularity optimizer. The Uniform Manifold Approximation and Projection (UMAP) was used to visualize the cell clusters in a two-dimensional space for cell annotation. Specifically, for identification of myeloid cell subgroups, we optimized the "FindClusters" function by setting the resolution to 1.1. Functional annotation of each myeloid subgroup was performed with the single-sample Gene Set Enrichment Analysis (ssGSEA) method, using gene sets collected from The Molecular Signatures Database (MSigDB). Cell–cell communication was analyzed with the ‘CellPhoneDB’ package in python v3.7, the interaction was defined as the number of significant (*p*-value < 0.01) interacting gene pairs. The trajectory analysis for myeloid cells was performed with ‘monocle3’ package under default parameters. The relevant enriched transcription factors and protein kinases were analyzed by eXpression2Kinases (X2K). Visualizations were generated and arranged in the ‘ggplot2’ package.

### Bulk-sequencing data acquisition

Data for The Cancer Genome Atlas (TCGA) cohort [27] and Molecular Taxonomy of Breast Cancer International Consortium (METABRIC) cohort [28] were acquired from cBioportal (http://www.cbioportal.org/) and the GDC Legacy Archive (https://portal.gdc.cancer.gov/legacy-archive). 1076 patients from the TCGA dataset and 1904 patients from the METABRIC dataset were recruited in this study, missing data were processed as in the original publications. Data for the National Cancer Center (NCC) cohort was generated in two independent centers (National cancer center and the Fourth Hospital of Hebei Medical University). Briefly, surgically resected samples from patients diagnosed with HR (hormone receptor)-positive early-stage BRCA from Jan. 2007 to Dec. 2012 in two independent centers (National cancer center and the Fourth Hospital of Hebei Medical University) were collected. Exclusion criteria were: (1) patients currently or previously diagnosed with other malignancies; (2) inadequate tissue samples or missing clinical data. The final NCC cohort for analysis comprised 219 primary BRCA patients. Patients were followed up for a minimum of 5 years for disease recurrence and mortality status, their clinical data were extracted from relevant medical records. The I-SPY2-990 RNA-seq data of 987 patients from 10 arms of the neoadjuvant I-SPY2 TRIAL for aggressive early-stage breast cancer were collected from the Gene Expression Omnibus under accession number GSE194040.

### Tumor-infiltrating myeloid cells inference

To infer the abundance of tumor-infiltrating myeloid cells in bulk-seq data, we used CIBERSORTx to perform cross-platform deconvolution. The expression matrix consisting of highly variable genes across each myeloid cell subset was extracted from the Seurat object, and a signature matrix was constructed, which was subsequently used as the template to impute myeloid abundance in bulk-seq data. The deconvolution process was conducted in absolute mode to allow further computation of relative abundance and diversity.

### Myeloid diversity assessment

The proportion of each subtype of myeloid cell was calculated from their abundance divided by the sum, the diversity indices was then used to assess myeloid diversity across each sample. The indices were computed with the following equations:

$$Shannon\,\, index= -\sum_{i=1}^{n}({P}_{i}*\mathrm{ln}\left({P}_{i}\right))$$

$$Gini-Simpson\,\, index= 1-\sum_{i=1}^{n}({{P}_{i}}^{2})$$

$$Pielou\,\, index= \frac{Shannon\,\, index}{\mathrm{ln}n}$$

### Survival analysis

To evaluate the impact of myeloid diversity on clinical outcomes of breast cancer patients, we used the Cox proportional hazard model and log-rank test to analyze the overall survival (OS) and progression-free survival (PFS) in different cohorts. OS is defined as the time from surgery to death, regardless of disease recurrence, PFS is defined as the time from surgery to disease progression or death from any cause. The results of the log-rank test were depicted as Kaplan–Meier plots, and the results of the Cox model were shown as hazard ratio (HR). All survival analyses were performed with ‘survival’ and ‘survminer’ packages.

### Cancer-Immunity cycle analysis

To assess the relationship between myeloid diversity and anti-tumor immunity, we used the pre-defined gene sets from the Cancer-Immunity Cycle server to perform ssGSEA analysis. The gene sets conceptualized anti-tumor immunity as a series of stepwise events including releasing of cancer antigens, antigen presentation, priming and activation, immune cell recruiting, and immune cell infiltration.

### Somatic mutation analysis

The somatic mutation data were analyzed with the ‘maftools’ package in R. High and low diversity patients were defined as patients whose myeloid diversity indices were among the top and bottom quarter of all patients in the cohort. Differentially presented mutations were detected by the chi-square test and filtered by a *p*-value lower than 0.01. Co-occurrence of mutations was analyzed with the ‘somaticInteractions’ function using default parameters.

### Weighted gene co-expression network analysis (WGCNA)

To delineate genes that were highly related to myeloid diversity, we used WGCNA to construct a co-expression network. Different co-expressed gene modules were clustered from the network, using the one-step method, the power for the soft threshold was set to 8, and all other parameters were kept at default. Next, the eigenvalues of each gene module were calculated and tested for correlation with myeloid diversity. Genes from the module with the highest correlation coefficient were extracted for further analysis.

### Predictive model construction

To identify genes that are most relevant to myeloid diversity from previously extracted WGCNA module, a machine learning model was trained, and the importance of each feature was calculated using ‘caret’ package. Data were scaled and centered before training the model, 70% of the preprocessed data were randomly included in the training set while the rest were used as the validation set. The random forest method was selected for model fitting, the hyperparameters were tuned with the ‘traincontrol’ function. For development of a surrogate model, the genes with high importance from machine learning models trained in TCGA and NCC cohorts were screened to exclude multi-collinearity (*r* < 0.75) and then combined to fit a linear regression model. The same linear regression model was then tested in METABRIC cohort for external validation. Different models were compared using the Cox regression model and correlation analysis.

### Statistical analysis

Two-tailed Wilcoxon rank-sum test was used to compared gene expression between normal and tumor groups, Spearman’s rank correlation test was to test for correlations between variables. The logistic regression was used to determine relationship between myeloid diversity and neoadjuvant drug response. Unless otherwise specified, the significance level for all tests was defined as a *p*-value < 0.05.

## Supplementary Information

**Additional file 1**. Supplementary Tables and Figures.

## Data Availability

Resources of all published data in this manuscript were available as described in the Material and Methods. The NCC cohort datasets generated during and/or analyzed during the current study are available from https://ngdc.cncb.ac.cn/ under the accession number of HRA001039.

## Abbreviations

TME: Tumor microenvironment
DCs: Dendritic cells
BRCA: Breast cancer
ICIs: Immune checkpoint inhibitors
MDSCs: Myeloid-derived suppressor cells
TCGA: The cancer genome atlas
METABRIC: Molecular Taxonomy of Breast Cancer International Consortium
NCC: National Cancer Center
OS: Overall survival
PFS: Progression-free survival
HR: Hazard ratio
DEGs: Differentially expressed genes
ssGSEA: Single-sample Gene Set Enrichment Analysis
WGCNA: Weighted gene co-expression network analysis
TMB: Tumor mutation burden
OXPHOS: Oxidative phosphorylation
