# Supplementary material for: Infiltrating myeloid cell diversity determines oncological characteristics and clinical outcomes in breast cancer
Source: Breast Cancer Res. 2023 Jun 7;25:63. doi: 10.1186/s13058-023-01669-6 (PMC10246424; doi:10.1186/s13058-023-01669-6)
Supplement: Supplementary file 1 — Additional file 1. Supplementary Tables and Figures. [file 13058_2023_1669_MOESM1_ESM.docx]

**Supplemental Table 1 Comparison of 3 different diversity indices by Cox regression.**

| Index type | Hazard ratio (HR) | Lower 95% CI | Upper 95% CI | p-value | Cohort | Survival data |
| --- | --- | --- | --- | --- | --- | --- |
| Gini-Simpson | 0.1799 | 0.0657 | 0.4924 | 0.0008 | TCGA | OS |
| Shannon | 0.1619 | 0.0528 | 0.4961 | 0.0014 | TCGA | OS |
| Pielou | 0.1619 | 0.0528 | 0.4961 | 0.0014 | TCGA | OS |
| Gini-Simpson | 0.2334 | 0.0841 | 0.6479 | 0.0052 | TCGA | PFS |
| Shannon | 0.2369 | 0.0763 | 0.7356 | 0.0127 | TCGA | PFS |
| Pielou | 0.2369 | 0.0763 | 0.7356 | 0.0127 | TCGA | PFS |
| Gini-Simpson | 0.5611 | 0.3721 | 0.8461 | 0.0058 | METABRIC | OS |
| Shannon | 0.6025 | 0.4215 | 0.8612 | 0.0054 | METABRIC | OS |
| Pielou | 0.6025 | 0.4215 | 0.8612 | 0.0054 | METABRIC | OS |
| Gini-Simpson | 0.4681 | 0.2840 | 0.7715 | 0.0029 | METABRIC | PFS |
| Shannon | 0.4897 | 0.3181 | 0.7539 | 0.0012 | METABRIC | PFS |
| Pielou | 0.4897 | 0.3181 | 0.7539 | 0.0012 | METABRIC | PFS |
| Gini-Simpson | 0.1884 | 0.0457 | 0.7769 | 0.0209 | NCC | PFS |
| Shannon | 0.2031 | 0.0547 | 0.7543 | 0.0173 | NCC | PFS |
| Pielou | 0.2031 | 0.0547 | 0.7543 | 0.0173 | NCC | PFS |

**Supplemental Table 2 Multivariate Cox regression of myeloid diversity and demographic data in TCGA cohort.**

| Overall survival (OS) | Hazard ratio (HR) | Lower 95% CI | Upper 95% CI | p-value |
| --- | --- | --- | --- | --- |
| Myeloid diversity | 0.3124 | 0.1541 | 0.6336 | 0.0013 |
| Age of diagnosis | 1.0321 | 1.0193 | 1.0450 | 0.0000 |
| Asian | 1.0000 |  |  |  |
| Black | 1.0486 | 0.3156 | 3.4837 | 0.9382 |
| Other races | 1.0310 | 0.2629 | 4.0437 | 0.9651 |
| White | 0.8629 | 0.2707 | 2.7506 | 0.8031 |
|  |  |  |  |  |
| Progression-free survival (PFS) |  |  |  |  |
| Myeloid diversity | 0.3945 | 0.1936 | 0.8041 | 0.0105 |
| Age of diagnosis | 1.0026 | 0.9899 | 1.0154 | 0.6946 |
| Asian | 1.0000 |  |  |  |
| Black | 0.9072 | 0.3483 | 2.3633 | 0.8420 |
| Other races | 0.8087 | 0.2538 | 2.5771 | 0.7195 |
| White | 0.7243 | 0.2924 | 1.7941 | 0.4858 |

**Supplemental Table 3 Multivariate Cox regression results of myeloid diversity and breast cancer subtypes in TCGA cohort.**

| Overall survival (OS) | Hazard ratio (HR) | Lower 95% CI | Upper 95% CI | p-value |
| --- | --- | --- | --- | --- |
| Myeloid diversity | 0.2553 | 0.1187 | 0.5489 | 0.0005 |
| Subtype_Normal_like | 1.0000 |  |  |  |
| Subtype_LumA | 0.5301 | 0.2409 | 1.1667 | 0.1148 |
| Subtype_LumB | 0.8113 | 0.3532 | 1.8639 | 0.6222 |
| Subtype_Her2 | 1.1860 | 0.4827 | 2.9141 | 0.7100 |
| Subtype_Basal | 0.5222 | 0.2202 | 1.2380 | 0.1402 |
|  |  |  |  |  |
| Progression-free survival (PFS) |  |  |  |  |
| Myeloid diversity | 0.3483 | 0.1628 | 0.7453 | 0.0066 |
| Subtype_Normal_like | 1.0000 |  |  |  |
| Subtype_LumA | 0.5859 | 0.2661 | 1.2902 | 0.1844 |
| Subtype_LumB | 0.6559 | 0.2792 | 1.5407 | 0.3330 |
| Subtype_Her2 | 1.2591 | 0.5118 | 3.0979 | 0.6159 |
| Subtype_Basal | 0.7215 | 0.3091 | 1.6838 | 0.4503 |

**Supplemental Table 4 Multivariate Cox regression of myeloid diversity and demographic data in METABRIC cohort**

| Overall survival (OS) | Hazard ratio (HR) | Lower 95% CI | Upper 95% CI | p-value |
| --- | --- | --- | --- | --- |
| Myeloid diversity | 0.6497 | 0.4772 | 0.8846 | 0.0062 |
| Pre-menopause | 1.0000 |  |  |  |
| Post-menopause | 1.8107 | 1.5219 | 2.1543 | <0.0001 |
| Chemotherapy |  |  |  |  |
| No | 1.0000 |  |  |  |
| Yes | 1.6013 | 1.3598 | 1.8858 | <0.0001 |
| Endocrinotherapy |  |  |  |  |
| No | 1.0000 |  |  |  |
| Yes | 1.2237 | 1.0763 | 1.3913 | 0.0021 |
| Radiotherapy |  |  |  |  |
| No | 1.0000 |  |  |  |
| Yes | 0.7771 | 0.6868 | 0.8793 | 0.0001 |
|  |  |  |  |  |
| Progression-free survival (PFS) |  |  |  |  |
| Myeloid diversity | 0.6252 | 0.4321 | 0.9047 | 0.0127 |
| Pre-menopause | 1.0000 |  |  |  |
| Post-menopause | 1.0313 | 0.8607 | 1.2356 | 0.7385 |
| Chemotherapy |  |  |  |  |
| No | 1.0000 |  |  |  |
| Yes | 1.8290 | 1.5323 | 2.1833 | <0.0001 |
| Endocrinotherapy |  |  |  |  |
| No | 1.0000 |  |  |  |
| Yes | 1.0712 | 0.9202 | 1.2469 | 0.3748 |
| Radiotherapy |  |  |  |  |
| No | 1.0000 |  |  |  |
| Yes | 0.8986 | 0.7731 | 1.0446 | 0.1640 |

**Supplemental Table 5 Multivariate Cox regression results of myeloid diversity and breast cancer subtypes in METABRIC cohort.**

| Overall survival (OS) | Hazard ratio (HR) | Lower 95% CI | Upper 95% CI | p-value |
| --- | --- | --- | --- | --- |
| Myeloid diversity | 0.6741 | 0.4936 | 0.9206 | 0.0131 |
| Subtype_Normal_like | 1.0000 |  |  |  |
| Subtype_LumA | 0.8874 | 0.6924 | 1.1373 | 0.3454 |
| Subtype_LumB | 1.3368 | 1.0387 | 1.7205 | 0.0242 |
| Subtype_Her2 | 1.4577 | 1.1066 | 1.9202 | 0.0073 |
| Subtype_Basal | 0.9605 | 0.7374 | 1.2511 | 0.7652 |
|  |  |  |  |  |
| Progression-free survival (PFS) |  |  |  |  |
| Myeloid diversity | 0.6029 | 0.4137 | 0.8788 | 0.0085 |
| Subtype_Normal_like | 1.0000 |  |  |  |
| Subtype_LumA | 0.6965 | 0.5239 | 0.9261 | 0.0128 |
| Subtype_LumB | 1.2085 | 0.9088 | 1.6070 | 0.1927 |
| Subtype_Her2 | 1.3290 | 0.9734 | 1.8144 | 0.0734 |
| Subtype_Basal | 0.8873 | 0.6590 | 1.1948 | 0.4310 |

**Supplemental Table 6 Multivariate Cox regression of myeloid diversity and demographic data in NCC cohort**

| Progression-free survival (PFS) | Hazard ratio (HR) | Lower 95% CI | Upper 95% CI | p-value |
| --- | --- | --- | --- | --- |
| Myeloid diversity | 0.1219 | 0.0197 | 0.7548 | 0.0237 |
| Age of diagnosis | 1.0106 | 0.9876 | 1.0341 | 0.3699 |
| Surgery type |  |  |  |  |
| Mastectomy | 1.0000 |  |  |  |
| Lumpectomy | 1.1597 | 0.6269 | 2.1452 | 0.6369 |
| Endocrinotherapy |  |  |  |  |
| No | 1.0000 |  |  |  |
| Yes | 1.2502 | 0.4481 | 3.4881 | 0.6696 |

**Supplemental Table 7 Multivariate Cox regression results of myeloid diversity and breast cancer subtypes in METABRIC cohort.**

| Progression-free survival (PFS) | Hazard ratio (HR) | Lower 95% CI | Upper 95% CI | p-value |
| --- | --- | --- | --- | --- |
| Myeloid diversity | 0.0184 | 0.0005 | 0.6329 | 0.0269 |
| Subtype_LumA | 1.0000 |  |  |  |
| Subtype_LumB | 1.0516 | 0.6488 | 1.7046 | 0.8381 |

**Supplemental Table 8 Univariate Cox regression results of myeloid diversity and cell-type frequencies in 3 cohorts**

| Term | Hazard ratio (HR) | Lower 95% CI | Upper 95% CI | p-value | Survival data | Cohort |
| --- | --- | --- | --- | --- | --- | --- |
| Myeloid diversity | 0.3191 | 0.1581 | 0.6442 | 0.0014 | OS | TCGA |
| pDC | 0.8979 | 0.8382 | 0.9617 | 0.0021 | OS | TCGA |
| Mac_CXCL8 | 0.9810 | 0.9685 | 0.9937 | 0.0035 | OS | TCGA |
| Mac_APOE | 0.9679 | 0.9457 | 0.9906 | 0.0059 | OS | TCGA |
| proDC | 0.9244 | 0.8714 | 0.9805 | 0.0089 | OS | TCGA |
| cDC_LAMP3 | 0.9025 | 0.8264 | 0.9856 | 0.0224 | OS | TCGA |
| Mac_CXCL10 | 0.9783 | 0.9559 | 1.0013 | 0.0640 | OS | TCGA |
| Mac_FABP5 | 1.0052 | 0.9997 | 1.0107 | 0.0652 | OS | TCGA |
| Mono_S100A9 | 1.0689 | 0.9616 | 1.1881 | 0.2170 | OS | TCGA |
| Cycling_cells | 0.9974 | 0.9909 | 1.0039 | 0.4249 | OS | TCGA |
| Mac_CD14 | 1.0046 | 0.9918 | 1.0175 | 0.4816 | OS | TCGA |
| Mac_LYVE1 | 0.9964 | 0.9858 | 1.0071 | 0.5052 | OS | TCGA |
| Mac_SPP1 | 0.9974 | 0.9803 | 1.0148 | 0.7671 | OS | TCGA |
| cDC_CD1C | 1.0036 | 0.9349 | 1.0773 | 0.9216 | OS | TCGA |
| Mac_IL1B | 0.0001 | 0.0000 | Inf | 0.9941 | OS | TCGA |
| Mac_CCL4 | 0.0161 | 0.0000 | Inf | 0.9952 | OS | TCGA |
|  |  |  |  |  |  |  |
| pDC | 0.9072 | 0.8481 | 0.9705 | 0.0046 | PFS | TCGA |
| Mac_CXCL8 | 0.9832 | 0.9707 | 0.9960 | 0.0101 | PFS | TCGA |
| Myeloid diversity | 0.4051 | 0.1990 | 0.8248 | 0.0127 | PFS | TCGA |
| proDC | 0.9349 | 0.8821 | 0.9909 | 0.0232 | PFS | TCGA |
| Mac_LYVE1 | 0.9880 | 0.9771 | 0.9991 | 0.0346 | PFS | TCGA |
| cDC_LAMP3 | 0.9171 | 0.8432 | 0.9975 | 0.0435 | PFS | TCGA |
| Mac_CXCL10 | 0.9816 | 0.9602 | 1.0034 | 0.0970 | PFS | TCGA |
| Mac_APOE | 0.9907 | 0.9778 | 1.0036 | 0.1579 | PFS | TCGA |
| Mac_FABP5 | 1.0029 | 0.9971 | 1.0087 | 0.3310 | PFS | TCGA |
| Mac_CD14 | 1.0035 | 0.9907 | 1.0165 | 0.5904 | PFS | TCGA |
| Cycling_cells | 0.9986 | 0.9921 | 1.0052 | 0.6858 | PFS | TCGA |
| Mac_SPP1 | 0.9964 | 0.9787 | 1.0143 | 0.6898 | PFS | TCGA |
| Mono_S100A9 | 1.0252 | 0.9039 | 1.1628 | 0.6984 | PFS | TCGA |
| cDC_CD1C | 1.0073 | 0.9389 | 1.0808 | 0.8388 | PFS | TCGA |
| Mac_CCL4 | 0.0148 | 0.0000 | Inf | 0.9945 | PFS | TCGA |
| Mac_IL1B | 0.0001 | 0.0000 | Inf | 0.9947 | PFS | TCGA |
|  |  |  |  |  |  |  |
| proDC | 76.1173 | 4.9544 | 1169.4307 | 0.0019 | OS | METABRIC |
| Myeloid diversity | 0.6474 | 0.4765 | 0.8797 | 0.0054 | OS | METABRIC |
| Mac_FABP5 | 1.5856 | 1.1267 | 2.2315 | 0.0082 | OS | METABRIC |
| Mac_CXCL8 | 0.7647 | 0.5637 | 1.0374 | 0.0847 | OS | METABRIC |
| Cycling_cells | 1.5113 | 0.9085 | 2.5140 | 0.1117 | OS | METABRIC |
| Mac_CD14 | 2.4073 | 0.6387 | 9.0733 | 0.1944 | OS | METABRIC |
| cDC_LAMP3 | 0.2438 | 0.0161 | 3.6888 | 0.3086 | OS | METABRIC |
| Mono_S100A9 | 0.3278 | 0.0338 | 3.1747 | 0.3356 | OS | METABRIC |
| pDC | 0.2865 | 0.0201 | 4.0804 | 0.3564 | OS | METABRIC |
| cDC_CD1C | 0.6883 | 0.2887 | 1.6413 | 0.3996 | OS | METABRIC |
| Mac_SPP1 | 1.3490 | 0.6107 | 2.9801 | 0.4591 | OS | METABRIC |
| Mac_CXCL10 | 0.9280 | 0.6313 | 1.3641 | 0.7038 | OS | METABRIC |
| Mac_APOE | 0.9425 | 0.6927 | 1.2824 | 0.7063 | OS | METABRIC |
| Mac_CCL4 | 1.1019 | 0.5489 | 2.2122 | 0.7849 | OS | METABRIC |
| Mac_LYVE1 | 0.9502 | 0.5435 | 1.6611 | 0.8577 | OS | METABRIC |
| Mac_IL1B | NA | NA | NA | NA | OS | METABRIC |
|  |  |  |  |  |  |  |
| Myeloid diversity | 0.5420 | 0.3743 | 0.7847 | 0.0012 | PFS | METABRIC |
| Mac_CXCL8 | 0.6169 | 0.4264 | 0.8924 | 0.0103 | PFS | METABRIC |
| proDC | 71.8357 | 2.6253 | 1965.6656 | 0.0114 | PFS | METABRIC |
| Mac_FABP5 | 1.4897 | 0.9912 | 2.2389 | 0.0552 | PFS | METABRIC |
| Mac_CCL4 | 0.4708 | 0.1985 | 1.1170 | 0.0875 | PFS | METABRIC |
| Cycling_cells | 1.4219 | 0.7730 | 2.6154 | 0.2577 | PFS | METABRIC |
| cDC_CD1C | 1.7810 | 0.6401 | 4.9550 | 0.2689 | PFS | METABRIC |
| Mac_LYVE1 | 0.7822 | 0.3995 | 1.5314 | 0.4736 | PFS | METABRIC |
| Mac_CD14 | 1.7906 | 0.3376 | 9.4975 | 0.4938 | PFS | METABRIC |
| Mac_SPP1 | 1.2468 | 0.4829 | 3.2188 | 0.6485 | PFS | METABRIC |
| pDC | 1.9851 | 0.0996 | 39.5650 | 0.6534 | PFS | METABRIC |
| Mac_APOE | 1.0463 | 0.7282 | 1.5033 | 0.8065 | PFS | METABRIC |
| cDC_LAMP3 | 1.2284 | 0.0691 | 21.8394 | 0.8886 | PFS | METABRIC |
| Mono_S100A9 | 1.2068 | 0.0868 | 16.7711 | 0.8886 | PFS | METABRIC |
| Mac_CXCL10 | 0.9980 | 0.6363 | 1.5652 | 0.9929 | PFS | METABRIC |
| Mac_IL1B | NA | NA | NA | NA | PFS | METABRIC |
|  |  |  |  |  |  |  |
| Mac_FABP5 | 5.1713 | 1.8550 | 14.4160 | 0.0017 | PFS | NCC |
| Myeloid diversity | 0.1144 | 0.0192 | 0.6815 | 0.0173 | PFS | NCC |
| pDC | 0.0274 | 0.0011 | 0.6735 | 0.0277 | PFS | NCC |
| proDC | 0.0278 | 0.0008 | 0.9743 | 0.0483 | PFS | NCC |
| cDC_LAMP3 | 0.0000 | 0.0000 | 2.1320 | 0.0683 | PFS | NCC |
| cDC_CD1C | 0.0928 | 0.0066 | 1.3072 | 0.0782 | PFS | NCC |
| Mac_CCL4 | 0.2668 | 0.0526 | 1.3521 | 0.1106 | PFS | NCC |
| Mac_CD14 | 0.2512 | 0.0385 | 1.6401 | 0.1490 | PFS | NCC |
| Cycling_cells | 2.5307 | 0.6494 | 9.8618 | 0.1809 | PFS | NCC |
| Mac_LYVE1 | 0.4424 | 0.1303 | 1.5019 | 0.1909 | PFS | NCC |
| Mono_S100A9 | 0.4257 | 0.0956 | 1.8954 | 0.2624 | PFS | NCC |
| Mac_CXCL10 | 2.1902 | 0.5273 | 9.0970 | 0.2805 | PFS | NCC |
| Mac_CXCL8 | 0.3767 | 0.0511 | 2.7752 | 0.3379 | PFS | NCC |
| Mac_APOE | 2.3912 | 0.1553 | 36.8127 | 0.5320 | PFS | NCC |
| Mac_SPP1 | 1.5388 | 0.1431 | 16.5515 | 0.7221 | PFS | NCC |
| Mac_IL1B | 0.4567 | 0.0039 | 53.3815 | 0.7470 | PFS | NCC |

**Supplemental Table 9 Univariate Cox regression results of myeloid diversity and cell-type frequencies in TCGA-PRAD cohort**

| Term | Hazard ratio (HR) | Lower 95% CI | Upper 95% CI | p-value | Survival data |
| --- | --- | --- | --- | --- | --- |
| cDC_LAMP3 | 0.0129 | 0.0002 | 1.0351 | 0.0518 | OS |
| Myeloid Diversity | 0.0114 | 0.0000 | 3.5363 | 0.1265 | OS |
| Mono_FCGR3A | 0.0150 | 0.0000 | 10.5324 | 0.2091 | OS |
| Mac_TNFSF10 | 0.6133 | 0.2857 | 1.3167 | 0.2098 | OS |
| Mac_IL6 | 0.0651 | 0.0003 | 13.1947 | 0.3135 | OS |
| Mac_IL1RN | 0.3159 | 0.0301 | 3.3172 | 0.3368 | OS |
| MDSC_CD14 | 0.2975 | 0.0214 | 4.1356 | 0.3666 | OS |
| cDC_CLEC9A | 1.9221 | 0.1144 | 32.2906 | 0.6499 | OS |
| Mac_NLRP3 | 0.4700 | 0.0149 | 14.8046 | 0.6679 | OS |
| Mac_FABP5 | 0.0000 | 0.0000 | Inf | 0.9943 | OS |
|  |  |  |  |  |  |
| cDC_LAMP3 | 0.1356 | 0.0387 | 0.4754 | 0.0018 | PFS |
| Myeloid Diversity | 0.1175 | 0.0168 | 0.8191 | 0.0307 | PFS |
| Mac_IL6 | 0.1899 | 0.0385 | 0.9372 | 0.0414 | PFS |
| MDSC_CD14 | 0.4962 | 0.2335 | 1.0543 | 0.0684 | PFS |
| Mac_TNFSF10 | 0.8470 | 0.6855 | 1.0466 | 0.1241 | PFS |
| Mac_FABP5 | 5.0053 | 0.5430 | 46.1369 | 0.1553 | PFS |
| cDC_CLEC9A | 1.2576 | 0.4364 | 3.6241 | 0.6713 | PFS |
| Mac_NLRP3 | 1.0698 | 0.4048 | 2.8275 | 0.8918 | PFS |
| Mono_FCGR3A | 0.8901 | 0.1559 | 5.0822 | 0.8957 | PFS |
| Mac_IL1RN | 0.9922 | 0.5057 | 1.9467 | 0.9819 | PFS |

**Supplemental Table 10 Model coefficients of the surrogate linear model**

| Term | Coefficient | std.error | statistic |
| --- | --- | --- | --- |
| (Intercept) | 2.3631 | 0.0062 | 381.2648 |
| GFPT2 | 0.0205 | 0.0082 | 2.4892 |
| CD27 | 0.0154 | 0.0225 | 0.6844 |
| C3 | 0.0472 | 0.0082 | 5.7884 |
| GMFG | -0.0465 | 0.0225 | -2.0629 |
| HLA.DPB1 | 0.0321 | 0.0097 | 3.3021 |

**Supplemental Table 11 Multivariate Cox regression of surrogate myeloid diversity in TCGA cohort**

| Overall survival (OS) | Hazard ratio (HR) | Lower 95% CI | Upper 95% CI | p-value |
| --- | --- | --- | --- | --- |
| Surrogate myeloid diversity | 0.1208 | 0.0085 | 1.7246 | 0.1192 |
| Age of diagnosis | 1.0314 | 1.0187 | 1.0443 | <0.001 |
| Asian | 1.0000 |  |  |  |
| Black | 1.1935 | 0.3599 | 3.9587 | 0.7724 |
| Other races | 1.1642 | 0.2965 | 4.5707 | 0.8275 |
| White | 1.0139 | 0.3180 | 3.2327 | 0.9814 |
|  |  |  |  |  |
| Progression-free survival (PFS) |  |  |  |  |
| Surrogate myeloid diversity | 0.0414 | 0.0024 | 0.7243 | 0.0292 |
| Age of diagnosis | 1.0019 | 0.9893 | 1.0147 | 0.7658 |
| Asian | 1.0000 |  |  |  |
| Black | 0.9851 | 0.3783 | 2.5649 | 0.9754 |
| Other races | 0.8791 | 0.2760 | 2.8000 | 0.8274 |
| White | 0.8239 | 0.3326 | 2.0408 | 0.6756 |

**Supplemental Table 12 Multivariate Cox regression of surrogate myeloid diversity in METABRIC cohort**

| Overall survival (OS) | Hazard ratio (HR) | Lower 95% CI | Upper 95% CI | p-value |
| --- | --- | --- | --- | --- |
| Surrogate myeloid diversity | 0.3376 | 0.1474 | 0.7729 | 0.0102 |
| Pre-menopause | 1.0000 |  |  |  |
| Post-menopause | 1.7772 | 1.4930 | 2.1155 | <0.0001 |
| Chemotherapy |  |  |  |  |
| No | 1.0000 |  |  |  |
| Yes | 1.6767 | 1.4231 | 1.9755 | <0.0001 |
| Endocrinotherapy |  |  |  |  |
| No | 1.0000 |  |  |  |
| Yes | 1.2310 | 1.0827 | 1.3996 | 0.0015 |
| Radiotherapy |  |  |  |  |
| No | 1.0000 |  |  |  |
| Yes | 0.7699 | 0.6804 | 0.8713 | <0.0001 |
| Progression-free survival (PFS) |  |  |  |  |
| Myeloid diversity | 0.1825 | 0.0670 | 0.4973 | 0.0009 |
| Pre-menopause | 1.0000 |  |  |  |
| Post-menopause | 1.0041 | 0.8372 | 1.2041 | 0.9650 |
| Chemotherapy |  |  |  |  |
| No | 1.0000 |  |  |  |
| Yes | 1.9299 | 1.6163 | 2.3043 | <0.0001 |
| Endocrinotherapy |  |  |  |  |
| No | 1.0000 |  |  |  |
| Yes | 1.0800 | 0.9277 | 1.2572 | 0.3213 |
| Radiotherapy |  |  |  |  |
| No | 1.0000 |  |  |  |
| Yes | 0.8900 | 0.7657 | 1.0346 | 0.1291 |

**Supplemental Table 13 Multivariate Cox regression of surrogate myeloid diversity in NCC cohort**

| Progression-free survival (PFS) | Hazard ratio (HR) | Lower 95% CI | Upper 95% CI | p-value |
| --- | --- | --- | --- | --- |
| Surrogate myeloid diversity | 0.0164 | 0.0005 | 0.5715 | 0.0233 |
| Age of diagnosis | 1.0097 | 0.9869 | 1.0330 | 0.4068 |
| Surgery type |  |  |  |  |
| Mastectomy | 1.0000 |  |  |  |
| Lumpectomy | 1.2423 | 0.6811 | 2.2658 | 0.4793 |
| Endocrinotherapy |  |  |  |  |
| No | 1.0000 |  |  |  |
| Yes | 1.2624 | 0.4534 | 3.5149 | 0.6556 |


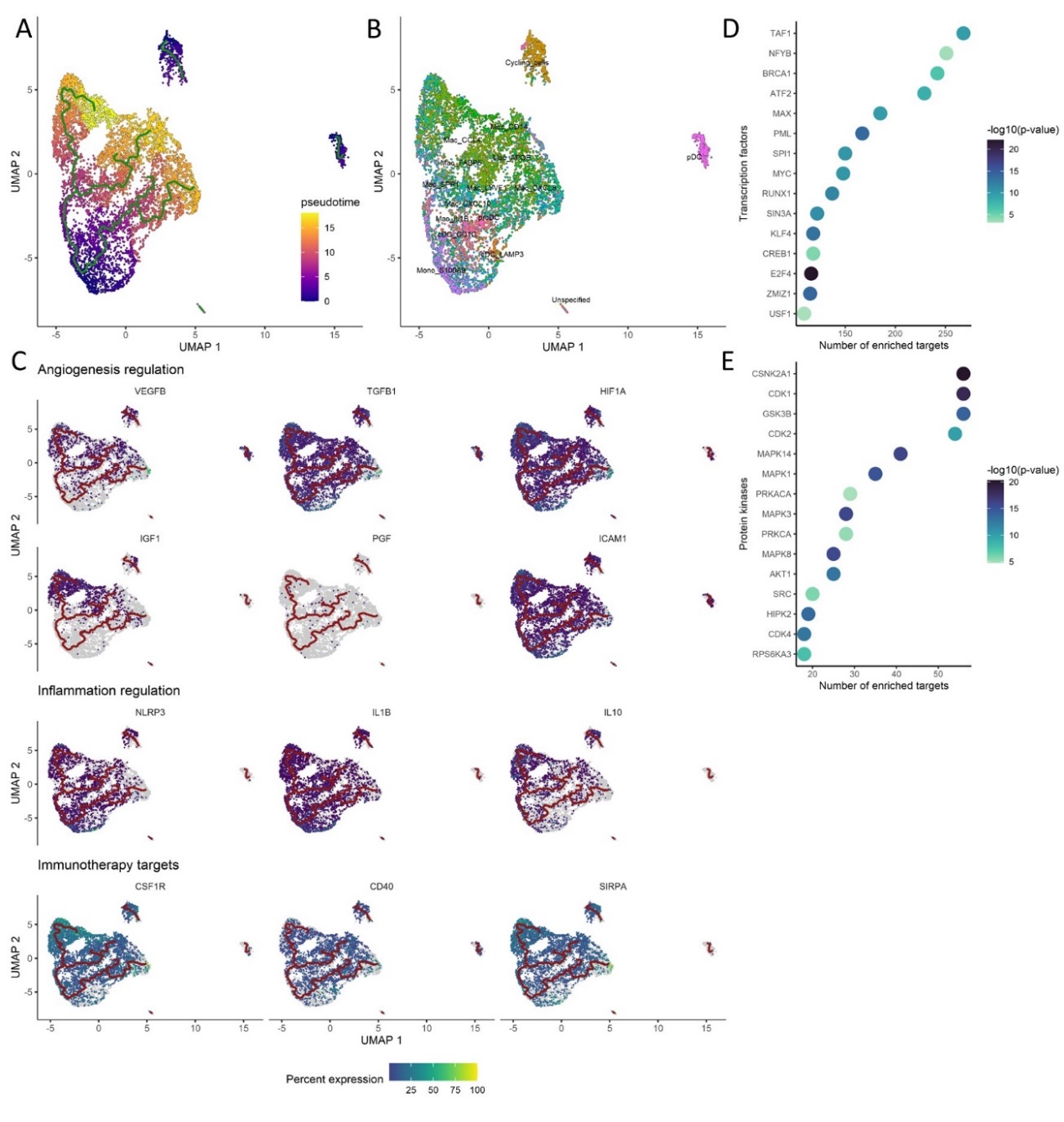


**Supplemental figure 1** (A, B) Distribution of breast cancer infiltrating myeloid cells along the pseudotime trajectory. (C) Representative genes regulating major myeloid pathways (angiogenesis, inflammation, tumor immunity), and their expression changes along the pseudotime trajectory. (D) Transcription factors enriched from differentially expressed genes along the pseudotime trajectory. (E) Protein kinases enriched from differentially expressed genes along the pseudotime trajectory.

**
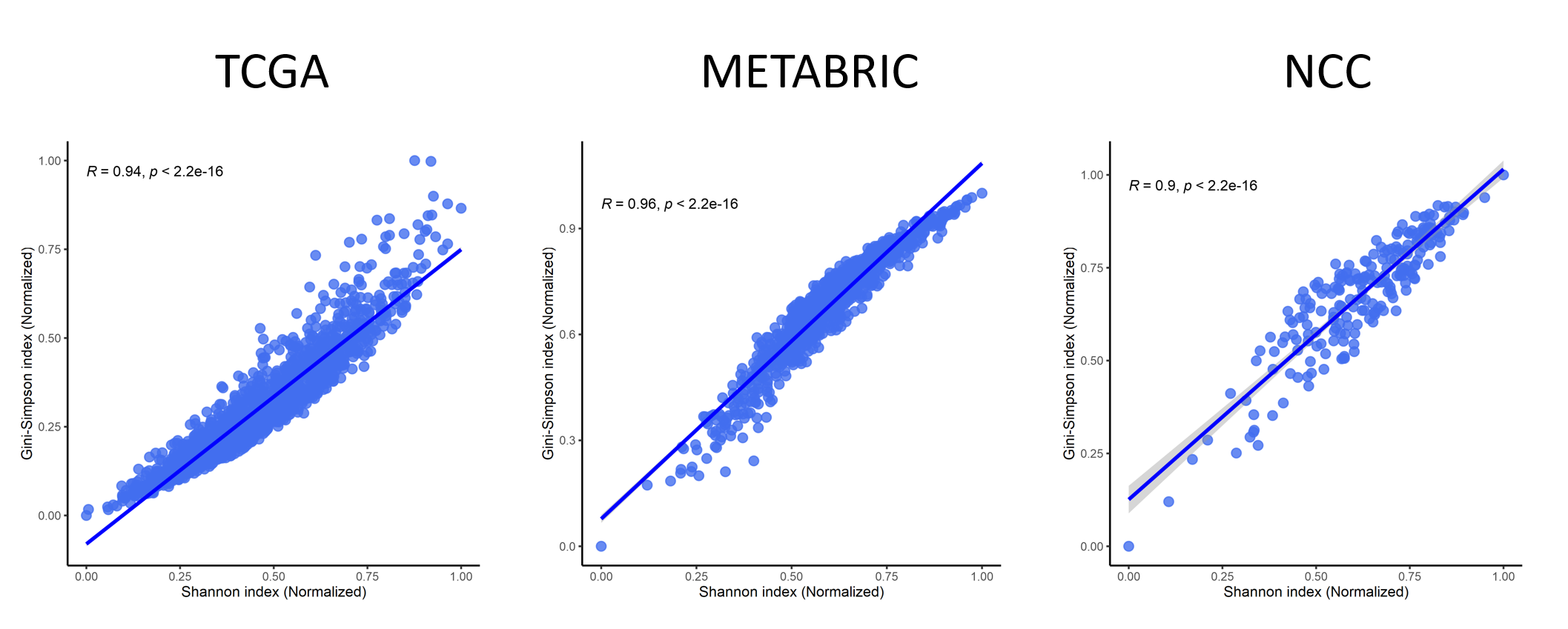
**

**Supplemental figure 2** (A) Scatter plot showing the correlation between Shannon index and Gini-Simpson index in the TCGA cohort (x axis represents normalized Shannon index, y axis represents normalized Gini-Simpson index). (B) Scatter plot showing the correlation between Shannon index and Gini-Simpson index in the METABRIC cohort. (C) Scatter plot showing the correlation between Shannon index and Gini-Simpson index in the NCC cohort.

**
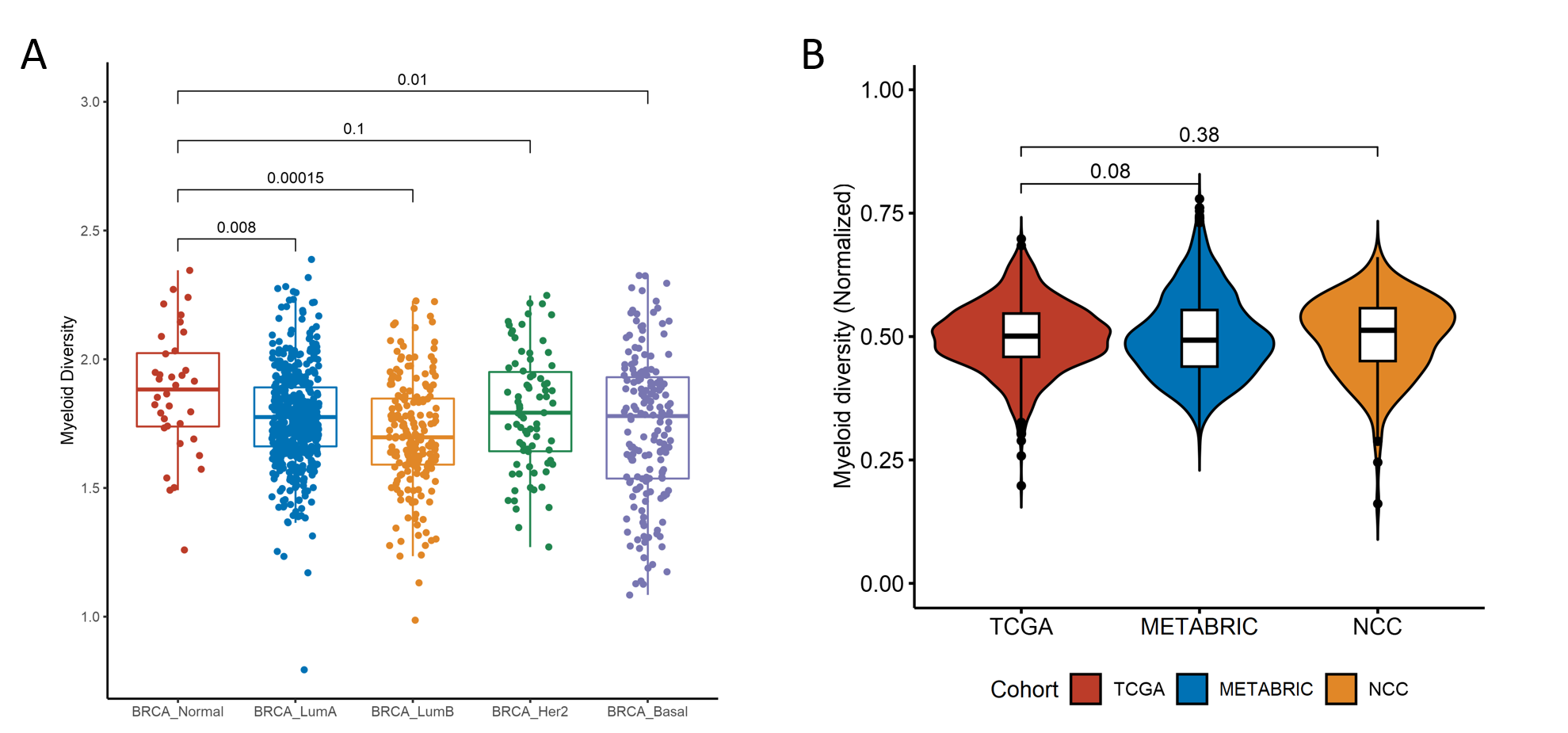
**

**Supplemental figure 3** (A) Myeloid diversity indices in different subtypes of breast cancer from the TCGA cohort (colors represent different subtypes, the Wilcoxon test was used to compare groups). (B) Violin and boxplot showing the distribution of normalized myeloid diversity index across the 3 cohorts (colors represent different cohorts, the Wilcoxon test was used to compare groups)

**
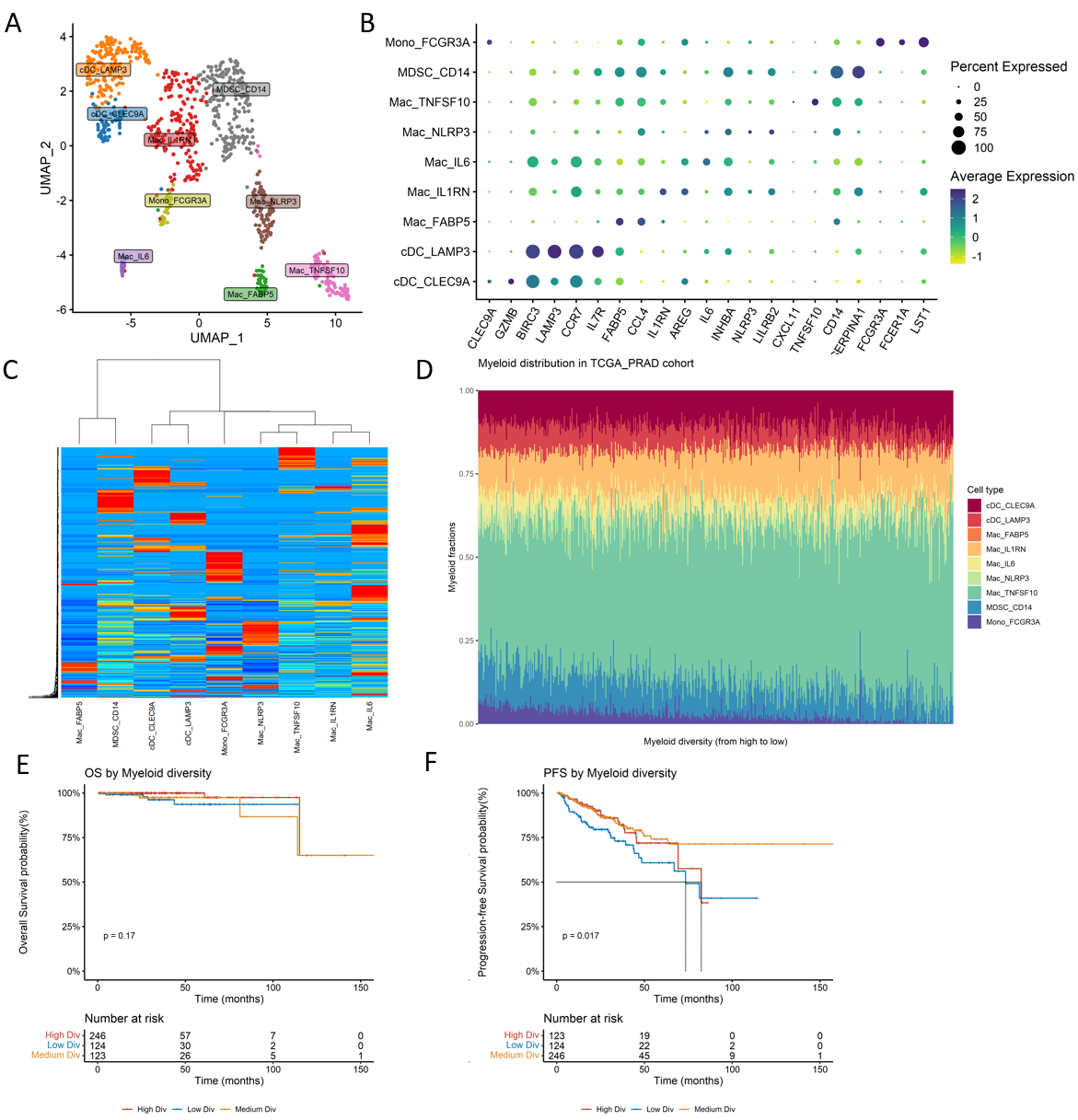
**

**Supplemental figure 4** (A) Projection of different subgroups of prostate cancer infiltrating myeloid cells in 2-dimensional UMAP space (axes represent dimensions, colors represent different cell subgroups). (B) Dot-plot of representative marker genes of each myeloid subgroup (y axis represents cell subgroups, x axis represents different genes, colors represent the average expression level of each gene, dot-size represents the expression percentage in each cell subtype). (C) Clustered signature matrix of prostate cancer infiltrating myeloid cells derived from single-cell analysis (y axis represents different genes, x axis represents cell subgroups, color represents gene expression levels (red: high expression; blue: low expression)). (D) Infiltrating myeloid cell distribution in the TCGA_PRAD cohort (y axis represents cell percentage in different colors, x axis represents myeloid diversity arranged in a decreasing manner). (E, F) Kaplan-Meier plot of survival analysis for patients with different levels of myeloid diversity in the TCGA_PRAD cohort (OS: overall survival, PFS: progression-free survival, colors represent different groups).

**
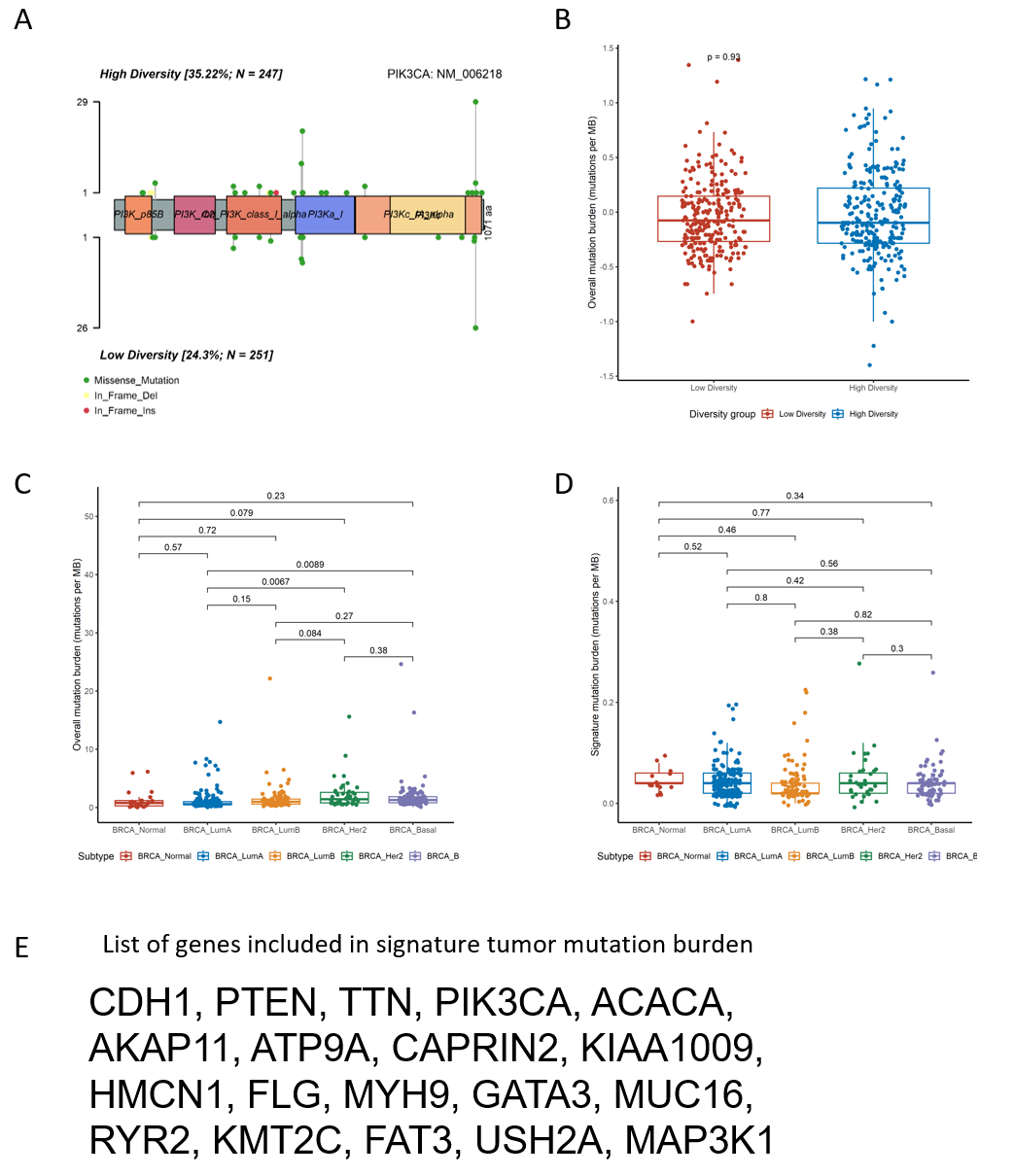
**

**Supplemental figure 5** (A) Schematic plot showing highly mutated domains in the protein structures of PIK3CA. (B) Boxplot of the overall tumor mutation burden between high and low diversity groups (colors represent different groups (red: low diversity group; blue: high diversity group)). (C, D) Boxplot of the overall and signature tumor mutation burden between different subtypes of breast cancer (colors represent different subtypes). (E) List of genes included in the signature tumor mutation burden. The Wilcoxon test was used to compare groups.
